# Small and Large Extracellular Vesicles Derived from Pleural Mesothelioma Cell Lines Offer Biomarker Potential

**DOI:** 10.3390/cancers15082364

**Published:** 2023-04-18

**Authors:** Tamkin Ahmadzada, Abhishek Vijayan, Fatemeh Vafaee, Ali Azimi, Glen Reid, Stephen Clarke, Steven Kao, Georges E. Grau, Elham Hosseini-Beheshti

**Affiliations:** 1 School of Medical Sciences, The University of Sydney, Camperdown, NSW 2006, Australia; 2 School of Biotechnology and Biomolecular Sciences, Faculty of Science, University of New South Wales, Sydney, NSW 2052, Australia; 3 UNSW Data Science Hub, University of New South Wales, Sydney, NSW 2052, Australia; 4 Westmead Clinical School, Faculty of Medicine and Health, The University of Sydney, Westmead, NSW 2145, Australia; 5 Centre for Cancer Research, The Westmead Institute for Medical Research, The University of Sydney, Westmead, NSW 2145, Australia; 6 Department of Dermatology, Westmead Hospital, Westmead, NSW 2145, Australia; 7 Department of Pathology, University of Otago, Dunedin 9016, New Zealand; 8 Department of Medical Oncology, Royal North Shore Hospital, Sydney, NSW 2065, Australia; 9 Department of Medical Oncology, Chris O’Brien Lifehouse, Sydney, NSW 2050, Australia; 10 Asbestos Diseases Research Institute, Sydney, NSW 2139, Australia; 11 The Sydney Nano Institute, The University of Sydney, Camperdown, NSW 2006, Australia

**Keywords:** extracellular vesicles, biomarkers, malignant pleural mesothelioma, oncosomes, microvesicles, exosomes

## Abstract

**Simple Summary:**

Pleural mesothelioma, a fatal thoracic cancer with one of the poorest survival rates of any cancer, is in urgent clinical need for biomarkers to aid early diagnosis, improve prognostication, and treatment options. Extracellular vesicles (EVs) have great potential as tumour biomarkers, however there are limited studies so far on their role in mesothelioma. We aimed to characterize different classes of EV derived from different mesothelioma cell lines. We provided a comprehensive proteomic database of cancer associated proteins in EVs that can offer new targets for future biomarker studies in pleural mesothelioma. We have also demonstrated that different subtypes of EVs can be isolated, namely 10 K, 18 K, and 100 K, each carrying oncogenic cargo with biomarker potential for pleural mesothelioma. Major differences were found in the cargo between the three EV subtypes, which can help narrow the molecular targets for diagnostic, prognostic, and predictive biomarker studies.

**Abstract:**

Pleural mesothelioma, previously known as malignant pleural mesothelioma, is an aggressive and fatal cancer of the pleura, with one of the poorest survival rates. Pleural mesothelioma is in urgent clinical need for biomarkers to aid early diagnosis, improve prognostication, and stratify patients for treatment. Extracellular vesicles (EVs) have great potential as biomarkers; however, there are limited studies to date on their role in pleural mesothelioma. We conducted a comprehensive proteomic analysis on different EV populations derived from five pleural mesothelioma cell lines and an immortalized control cell line. We characterized three subtypes of EVs (10 K, 18 K, and 100 K), and identified a total of 4054 unique proteins. Major differences were found in the cargo between the three EV subtypes. We show that 10 K EVs were enriched in mitochondrial components and metabolic processes, while 18 K and 100 K EVs were enriched in endoplasmic reticulum stress. We found 46 new cancer-associated proteins for pleural mesothelioma, and the presence of mesothelin and PD-L1/PD-L2 enriched in 100 K and 10 K EV, respectively. We demonstrate that different EV populations derived from pleural mesothelioma cells have unique cancer-specific proteomes and carry oncogenic cargo, which could offer a novel means to extract biomarkers of interest for pleural mesothelioma from liquid biopsies.

## 1. Introduction

Pleural mesothelioma, previously known as malignant pleural mesothelioma or MPM, is a rare but fatal cancer that forms on the thin membrane that lines the lungs and thoracic cavity [1]. It continues to have one of the poorest survival rates of any cancer, with a median survival of typically less than a year after diagnosis for untreated patients [1,2] or 16 to 18 months for patients receiving treatment [3,4], and a five-year survival rate of around 7% [5]. The primary cause of pleural mesothelioma is exposure to asbestos fibres, a group of naturally occurring fibrous minerals with excellent physical and electrochemical insulating properties and a long history of industrial applications [5,6]. Despite a progressive ban on asbestos production and usage in over 50 countries worldwide since the 1980s, the incidence of pleural mesothelioma continues to rise due to the long latency period (20 to 50 years) between asbestos exposure and the development of the tumor [5,6,7]. As a result, pleural mesothelioma is common in the elderly population and in males with a history of occupational exposure to asbestos [5].

The diagnosis of pleural mesothelioma can be a complex process requiring multiple techniques, such as invasive tissue biopsy, cytological examination of the pleural fluid, and thoracoscopy, accompanied by immunohistochemistry staining on a wide panel of markers to differentiate pleural mesothelioma from other tumors [8]. Consequently, pleural mesothelioma is often diagnosed at an advanced stage and has a poor prognosis. Currently, the performance status and histological subtype are the only factors used as prognostic indicators [9,10], with three histologic subtypes validated in clinical practice. Epithelioid is the most common subtype of pleural mesothelioma, constituting up to two-thirds of cases, with a survival of 19 months [2,11]; sarcomatoid is the least common subtype with the worst survival of 4 to 6 months [11], and biphasic is a mixture of epithelioid and sarcomatoid subtypes, with the survival dependent on the ratio of epithelioid and sarcomatoid cells present in the tumor [11].

Until 2020, the treatment for pleural mesothelioma was limited to systemic combination chemotherapy, typically platinum or cisplatin and pemetrexed with or without bevacizumab, with only modest improvements in survival [9,10,12]. In recent years, immunotherapy using immune checkpoint inhibitors has transformed the treatment landscape of pleural mesothelioma. In October 2020, the US Food and Drug Administration approved the combination immunotherapy drugs, nivolumab (anti-programmed death-1 antibody) plus ipilimumab (anti-cytotoxic T-lymphocyte antigen-4 antibody), as first-line treatment for patients with unresectable pleural mesothelioma. This came following the results of the CHECKMATE-743 trial, which showed a median overall survival of 18.1 months for patients who received combination immunotherapy versus 14.1 months for patients who received chemotherapy [4]. A recent study with a minimum of three years of follow-up further showed that nivolumab plus ipilimumab continued to provide long-term survival benefit over chemotherapy, regardless of histology [13], indicating that immunotherapy is the new treatment modality for pleural mesothelioma. Nevertheless, not all patients benefit from immunotherapy, and although significant progress has been made for the treatment of pleural mesothelioma, there remains an urgent need for biomarkers and molecular targets that could help with early diagnosis, improve prognostication, help stratify patients to targeted treatments, and help select patients who might benefit from immunotherapy. There is also an important clinical need for non-invasive or minimally invasive techniques for detecting biomarkers without using invasive tissue biopsies.

During the formation and growth of the tumor, various components may be released into the body fluids due to apoptosis, necrosis, or active release of particles [14]. These include circulating tumor cells, circulating tumor DNA, circulating tumor RNA, and extracellular vesicles (EVs) [14]. These offer a means to identify biomarkers from liquid biopsies. EVs have demonstrated enormous potential as biomarkers for cancer, including for pleural mesothelioma. EVs are membrane-contained particles secreted by malignant and non-malignant cells to transport biological information from one cell to another and mediate intercellular communication [15,16,17,18]. Their cargo composition consists of different classes of proteins, lipids, and genetic material including DNA, RNA, mRNA, and miRNA [15,16,17,18]. EVs reported in the literature are typically classified according to their size and biogenesis; however, there is substantial overlap. Although there is currently no standardized nomenclature for different EV populations, the most common EV subtypes reported in the literature include small EVs (50–150 nm) [19], commonly called exosomes, formed from the endosomal biogenesis pathways and typically isolated using a centrifugal force ranging from 100,000× *g* to 200,000× *g*; larger-sized EVs (50–1000 nm), previously termed microparticles and later changed to microvesicles, formed by blebbing from the plasma membrane and typically isolated using a centrifugal force of 18,000× *g*; and apoptotic bodies (1–4 µm), which are shed from dying cells and are typically isolated using a centrifugal force of 2000–3000× *g* [15,19,20]. Recently, EVs containing oncogenic cargo have been characterized, named oncosomes (100–400 nm) and large oncosomes (1–10 µm), which are thought to be shed exclusively from cancer cells [21]. These so-called large oncosomes have been isolated using a centrifugal force of 10,000× *g* [15,20,21].

EVs circulate freely in almost all body fluids, including blood, urine, milk, and saliva [19], making them an ideal candidate for non-invasive or minimally invasive biopsies. Furthermore, EVs are encapsulated by a phospholipid membrane, which protects their contents from degradation, making them highly stable in body fluids [22]. Growing evidence suggests that the behavior and functional roles of EVs are specific, not random, which can help reveal the tumor-specific immune suppression mechanisms for individual patients [15,16,17,18]. These qualities make EVs ideal biomarker candidates for pleural mesothelioma over tissue-based biomarkers, due to the invasiveness of tissue biopsies, the difficulty in capturing the tumor heterogeneity of pleural mesothelioma and its subtypes from only a section of the tumor tissue, and the limited access to tissue samples from mostly elderly patients who are typically already at an advanced stage of disease and are usually poor candidates for surgery [14]. EVs have a major advantage of being available in body fluids, offering a novel pathway to perform liquid biopsies for pleural mesothelioma.

Our study had two main objectives. Firstly, we aimed to characterize and distinguish the proteomes of three subtypes of EVs derived from pleural mesothelioma cell lines using different centrifugal forces: large-sized EVs isolated at 10,000× *g* (10 K) and at 18,000× *g* (18 K), and then small-sized EVs isolated at 100,000× *g* (100 K). Secondly, we aimed to identify unique cancer-specific proteins that are contained within the different EV subtypes derived from pleural mesothelioma cell lines, which could offer a novel means to extract specific biomarkers of interest from liquid biopsies. To our knowledge, this is the first study that distinguishes three subtypes of EVs, and the first to characterize larger-sized EVs in pleural mesothelioma.

## 2. Materials and Methods

### 2.1. Cell Culture

Five mesothelioma cell lines and an immortalized human mesothelial cell line (Table 1) were cultured in growth medium consisting of Roswell Park Memorial Institute (RPMI) 1640 with L-glutamine and sodium bicarbonate (R8758; Sigma–Aldrich, St. Louis, MO, USA), supplemented with 10% fetal bovine serum (FBS) (F9423; Sigma–Aldrich, MO, USA). Cells were grown at 37 °C in a humidified atmosphere of 5% CO_2_. Cell viability was assessed using trypan blue exclusion methods (T8154; Sigma–Aldrich, MO, USA). Cell viability and cell counts were obtained using an automated cell counter (Countess II, Thermo Fisher Scientific, Waltham, MA, USA). 

### 2.2. EV Isolation

Each cell line was initially grown to 80% confluence in T25 flasks, and then passaged to a T75 flask. When cells reached 80% confluence in the T75 flask, each cell line was passaged to eleven T75 flasks, where ten flasks were maintained for EV isolation and one T75 flask was maintained to repeat another round of EV isolation. Once the cells reached 70% confluence, they were starved of FBS and kept in RPMI 1640 alone for 72 h. The conditioned media from all ten flasks were pooled in 50 mL centrifuge tubes and centrifuged at 2000× *g* for 10 min (4 °C) to remove protein aggregates and cell debris. The cleared medium was then concentrated using 100 KDa MWCO Amicon^®^ Ultra-15 Centrifugal Filter Units (UFC910096, Millipore, Billerica, MA, USA), by repeatedly performing centrifugation at 2000× *g* for 5 min at 4 °C until 2 mL of sample remained above the filter. The concentrated 2 mL samples were transferred to Eppendorf tubes and centrifuged at 10,000× *g* for 45 min at 4 °C to obtain our 10 K EV sample. The supernatant was transferred to new Eppendorf tubes. The new sample tubes were centrifuged at 18,000× *g* for 45 min at 4 °C to obtain our 18 K EV sample. The supernatant was then transferred to ultracentrifuge tubes. In the ultracentrifuge tubes, 200 µL of 30% sucrose-deuterium oxide (D_2_O) was deposited at the bottom of the tubes. The samples were centrifuged at 100,000× *g* for 60 min at 4 °C (Hitachi CP100NX ultracentrifuge, P50A3 rotor) to obtain our 100 K EV sample. Finally, the supernatant was removed, and the 100 K pellets were resuspended in the sucrose cushion. All pellets and supernatants were retained and stored at −80 °C. In total, four rounds of EV isolation were completed for each cell line to produce four biological replicates. In each round of EV isolation, two technical replicates were produced.

### 2.3. Protein Quantification

Protein lysates of cells and EVs were prepared via lysis in radioimmunoprecipitation assay (RIPA) buffer (89900; Thermo Fisher Scientific) and protease inhibitors (11836153001; cOmplete™, Mini Protease Inhibitor Cocktail, Roche Diagnostics, Mannheim, Germany). Protein concentrations were determined using the Pierce^™^ Bicinchoninic Acid (BCA) Protein Assay Kit (Thermo Fisher Waltham, MA, USA) according to the manufacturer’s instructions. Absorbance was measured at 562 nm using a spectrophotometer. Protein concentration was interpolated based on the constructed standard curves.

### 2.4. Nanoparticle Tracking Analysis

Nanoparticle tracking analysis (NTA) was performed using the NanoSight^™^ LM10 instrument (Malvern, Analytical), with the green laser (532 nm) to measure the number of particles and the particle size distribution of EV samples. The instrument temperature was set to 25 °C, the camera level was set to 11 and the detection threshold set to 2; however, the camera was manually focused where needed to optimize particle definition. Samples were diluted 1 in 500 with filtered and double distilled water (DDH_2_O) to a volume of 1 mL to maintain a concentration below 5 × 10^9^ particles/mL. For the first reading, an initial volume of 0.5 mL of the sample was injected into the sample viewing unit using a syringe. For the subsequent readings, samples were injected in 0.1 mL increments. Videos were captured in 5 repetitive cycles, with each cycle analyzing 0.1 mL of injected sample. The sample viewing unit was washed three times in between samples with DDH_2_O followed by 80% *v*/*v* ethanol. The movement of particles due to Brownian motion was recorded every 60 s at 30 frames per second. All NTA experiments were performed at room temperature.

### 2.5. Flow Cytometry

EV samples (10 K and 18 K only) were stained using the Annexin V-FITC kit (IM3614, Beckman Coulter, Brea, CA, USA) according to the manufacturer’s instructions. Briefly, in a 96-well plate with a round bottom, 20 µL of the EV sample was incubated with 1 µL of Annexin V and 2 µL of 10× concentrated binding buffer for 20 min in the dark. Samples were then suspended in 180 µL of 1× binding buffer solution, prepared with sterile water, to a final sample volume of 200 μL. Samples were transferred to flow tubes and the fluorescent signals were measured on the Gallios™ Flow Cytometer (Beckman Coulter, CA, US) using the 488 nm blue laser. Samples were measured over 120 s at a medium flow rate (37 μL/min). We used the protocol previously described by our group to define the gates and the different particle sizes for small and large EVs [23]. Data were analyzed using Kaluza software (v1.2; Beckman Coulter, Lane Cove, NSW, Australia).

### 2.6. Western Blotting

Protein samples were denatured by adding 2 × concentrate Laemmli sample buffer with 10% β-mercaptoethanol (S3401, Merck KGaA, Darmstadt, Germany) and heating to 95 °C for 5 min. Equal amounts of proteins (50 μg) were loaded and electrophoresed on 10% SDS-PAGE gels at 110 V for 90 min using Mini-Protean^®^ Tetra cells (Bio-Rad Laboratories, Hercules, CA, USA) filled with Tris/Glycine/SDS running buffer containing 25 mM Tris-HCl, 192 mM glycine, and 0.1% SDS (1610772, Bio-Rad Laboratories, Hercules, CA, USA). The electrophoresed proteins were transferred onto a nitrocellulose membrane (0.45 μm) at 110 V for 105 min using a transfer buffer consisting of Tris/Glycine buffer with 25 mM Tris and 192 mM glycine (1610771, Bio-Rad Laboratories, Hercules, CA, USA) and 20% methanol. Membranes were blocked in a blocking buffer containing 5% w/v non-fat dry milk in Tris buffer saline (TBS) plus tween 20 (TBST) (10 mM Tris; 100 mM NaCl; 0.1% Tween 20), for 1 h at room temperature on an orbital shaker. The membranes were incubated with a primary antibody (Table 2) in TBST overnight at 4 °C on an orbital shaker. Then, membranes were washed three times with TBST for 5 min each and incubated with a HRP-conjugated secondary antibody (Cell Signaling Technology, Danvers, MA, USA, 1:5000) for 1 h at room temperature. The immunoreactive blots were visualized using the Clarity^™^ Western ECL Substrate (1705060, Bio-Rad Laboratories, Hercules, CA, USA), and then imaged using the ChemiDoc Imaging System (Bio-Rad Laboratories, Hercules, CA, USA).

### 2.7. Transmission Electron Microscopy (TEM)

Representative samples from large and small EVs were used for imaging using TEM. Samples were adsorbed onto carbon copper 200 mesh grids (GSCU200C-50; 5 uL/grid, Proscitech, Thuringowa, QLD, Australia) and incubated in the dark for 1 min. Samples were then washed with water and then negatively stained with 2% Uranyl Acetate for 3 min at room temperature. Grids were air dried and TEM images were collected with the JOEL 1400 transmission electron microscope (JOEL, Tokyo, Japan) at 120 kV.

### 2.8. Mass Spectrometry (LC-MS/MS)

EVs were lysed in 6 M urea, 2 M thiourea, 100 mM HEPES buffer, pH 7.5 and then sonicated on ice for 10 min. Proteins (50 μg) for each sample were reduced with 10 mM DL-Dithiothreitol (DTT) for 30 min at room temperature. They were then alkylated with 25 mM iodoacetamide for 30 min at room temperature in the dark, and then quenched with DTT to a final concentration of 20 mM. Samples were diluted with 100 mM HEPES, and then digested with 1 μg of trypsin (Promega Sequencing Grade) overnight at 30 °C. Samples were then acidified with 1% trifluoroacetic acid (TFA) and desalted on HLB columns, Oasis 10 mg extraction cartridges (Waters™, Milford, MA, USA). Peptide separation was achieved by eluting with 50% acetonitrile (ACN) plus 0.1% TFA. Columns were washed three times with 5% ACN plus 0.1% TFA before and after loading the samples. Samples were analyzed on a Q Exactive HFX3 Mass Spectrometer (Thermo Fisher Scientific).

### 2.9. Proteomic Data Analysis

Raw data were processed using Proteome Discoverer^™^ (v2.2, Thermo Fisher Scientific, Waltham, MA, USA). The MS/MS spectra were searched using Mascot (Matrix Science, London, UK; version 2.4.0) against the UniProt human and contaminants databases (Human, May 2020; Contaminants, November 2018). The following parameters were applied: trypsin digestion, with up to two missed cleavages; variable modifications to carbamidomethyl (C), protein N-terminal acetylation, deamidation (NQ), and oxidation (M); precursor mass tolerance of 10 ppm; MS/MS tolerance of 0.05 Da, and minimum peptide length of 6. Spectral matches were validated using Percolator based on q-values with a maximum delta CN of 0.05 and false discovery rate (FDR) of 1%. Proteins were quantified using label-free quantification and grouped according to a strict parsimony principle. All data for proteomic analysis were retrieved using Proteome Discoverer software version 2.4.1.15 (Thermo Fisher Scientific, Bremen, Germany).

### 2.10. Statistical Analysis

Statistical analysis for NTA and flow cytometry was performed using GraphPad Prism 5.0 Software (GraphPad Software Inc., San Diego, CA, USA). Pooled data were used, and Mann-Whitney U tests were conducted for each comparative set of unpaired data. Effects were considered significant when *p* value was ≤0.05 (* *p* ≤ 0.05, ** *p* ≤ 0.01, *** *p* ≤ 0.001 and **** *p* ≤ 0.0001).

Statistical analysis for proteomics was conducted in R. Proteins with missing values in less than 75% of the replicates were retained. The remaining missing values were imputed using the missForest algorithm [24]. Non-human proteins and contaminant proteins were discarded. FilterByExpr from edgeR package in R was used to filter low abundance proteins. The resultant data were quantile normalized. For each sample, technical replicates were averaged; when the difference between protein abundances across technical replicates was >90% quantile across all proteins, the minimum abundance between the two technical replicates was used in lieu of averaging. The differential expression analysis was conducted via limma-voom [25] R package, and *p*-values were adjusted for multiple hypotheses testing using the Benjamini-Hochberg correction; the (s)adjusted *p*-value < 0.05 was considered to be significant. The UMAP (Uniform Manifold Approximation and Projection) algorithm was used for non-linear dimension reduction to produce 2-dimensional embeddings of each sample and generate 2D scatter plots to visualize how separable the classes under consideration are (e.g., EV subtypes). All the visualizations and analyses mentioned above were conducted in R. The visualizations were primarily created using ggplot and ComplexHeatmap [26,27]. Functional enrichment analysis for biological processes and cellular components was performed on the Proteome Discoverer software using the false discovery rate adjusted *p* value < 0.05. To better understand how changes in the proteome may affect mesothelium’s pathophysiology, we employed Ingenuity Pathway Analysis (IPA) bioinformatics tool (Ingenuity Systems, USA; version 23.0; release date: 17 January 2023; http://analysis.ingenuity.com) to retrieve information about cancer-related molecular pathways and biological functions predicted to be activated or inhibited by proteins significantly changed in the EV subtypes.

## 3. Results

### 3.1. Morphological Characteristics of Pleural Mesothelioma Cell Lines

**Figure 1:**
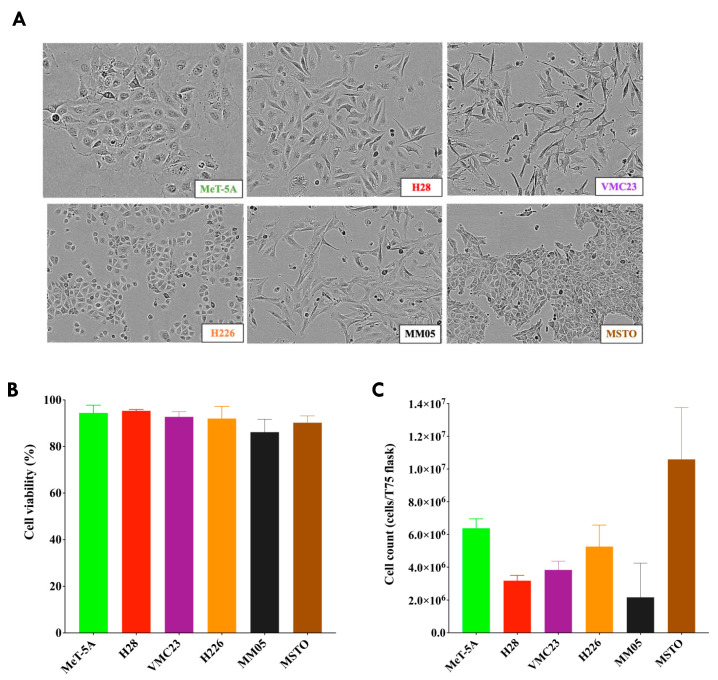
Mesothelioma cell lines. (**A**) Cell morphology monitored every 2 h via light microscopy using real time in vitro IncuCyte^®^ zoom imaging system; (**B**) Percentage of live cells recorded on a Countess live cell counter after the starvation period and just before EVs were isolated. (**C**) The number of cells in a T75 flask for each cell line after the starvation period and just before EVs were isolated, recorded on a Countess live cell counter. Data for cell viability and cell counts were calculated from an average of three T75 flasks for each cell line just before EV isolation.

Five pleural mesothelioma cell lines and one immortalized cell line were included in this study, as shown in Table 1. Figure 1A shows the morphology of the cell lines under the IncuCyte^®^ live-cell imaging equipment. Cells were grown in culture as a monolayer of adherent cells and exhibited varying cell morphologies. MeT-5A, H226, and MSTO displayed epithelial-like cell morphology with regular shaped and uniform cuboidal or ‘cobble-stone’ configuration, whereas H28, VMC23, and MM05 displayed fibroblast-like cell morphology. Cells derived from the same histological subtype of pleural mesothelioma did not show similar morphologies in culture. The percentage and number of live cells were counted on the day of EV isolation from three representative flasks. On average, each cell line had at least 80% viability on the day of EV isolation (Figure 1B), with more than 2 million live cells on average in each T75 flask (Figure 1C).

### 3.2. Characterization of 10 K, 18 K, and 100 K EVs

**Figure 2:**
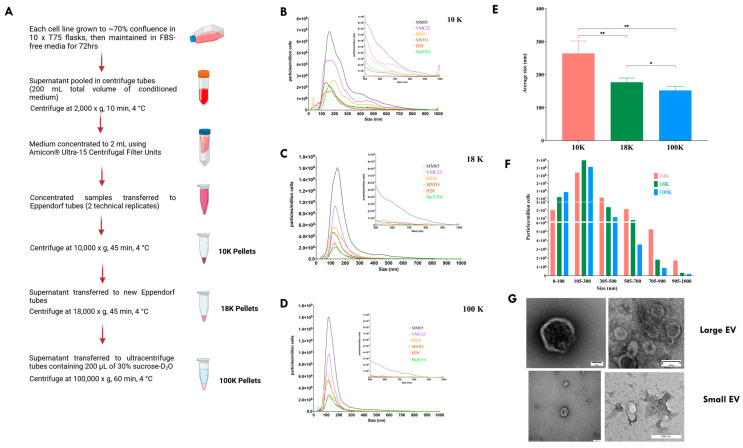
EV isolation, particle size distribution, and particle concentration. (**A**) Flowchart of the differential centrifugation and ultracentrifugation method used for EV isolation. (**B**) Size distribution and particle concentration profile of 10 K EV samples as measured using NTA. (**C**) Size distribution and particle concentration profile of 18 K EV samples as measured using NTA. (**D**) Size distribution and particle concentration profile of 100 K EV samples as measured using NTA. (**E**) Comparison of the average size of particles in 10 K, 18 K, and 100 K pellets, as measured using NTA. (**F**) Particle concentration of 10 K, 18 K, and 100 K according to size clusters, showing the dominant EV type in each size range, as measured using NTA. (**G**) Transmission electron microscopy showing cup-shaped EV morphology for large and small EVs. * *p* ≤ 0.05, ** *p* ≤ 0.01.

Figure 2A shows a schematic of the protocol used to isolate the three types of EVs in this study. EVs from pooled conditioned medium were isolated as described in Figure 2A to obtain 10 K, 18 K, and 100 K EV pellets. NTA profiles show that, overall, mesothelioma cell lines produced more EVs compared to the control cell line, MeT-5A (Figure 2B–D). There is also a consistent trend in the hierarchy of high to low EV production by mesothelioma cell lines: MM05 released the highest number of particles across 10 K, 18 K, and 100 K pellets, despite having the lowest cell count prior to EV isolation (Figure 1C), followed by VMC23, H226, and MSTO. H28 produced the lowest number of particles across all EV types and was comparable to the control cell line, MeT-5A, in terms of the number of particles released. The highest concentration of particles captured by NTA was in the size range of 100 to 300 nm across all EV types. On close inspection of the larger size range of the scale (500 nm–1 µm), 10 K EV pellets produced a relatively higher concentration of large-size particles (Figure 2B), 18 K EV pellets produced a relatively lower concentration of large-sized particles (Figure 2C), whereas 100 K EV pellets had the lowest concentration of large-sized particles (Figure 2D). The pooled average size of particles in each EV pellet was calculated (Figure 2E), showing 10 K EVs having a significantly higher average particle size compared to 18 K (*p* = 0.002) and 100 K (*p* = 0.002). Moreover, 18 K EV samples had a significantly higher average particle size compared to 100 K (*p* = 0.02). Overall, although each pellet contained particles of all sizes, as small as <100 nm and as large as 1 µm, the larger-sized particles (from 305 nm to 1 µm) consisted of mostly 10 K EVs, medium-sized particles (105–300 nm) consisted of mostly 18 K EVs, and small-sized particles (≤100 nm) consisted of mostly 100 K EVs (Figure 2F).

Representative EV samples were imaged using TEM (Figure 2G), showing the characteristic “cup-shaped” vesicles as a result of dehydration during sample preparation. It is apparent that large EV samples (10 K) contain a much higher concentration of EV population compared to the small EV samples (100 K). Furthermore, EVs contained in the 10 K pellets contain smaller-sized vesicles, consistent with our NTA results.

**Figure 3:**
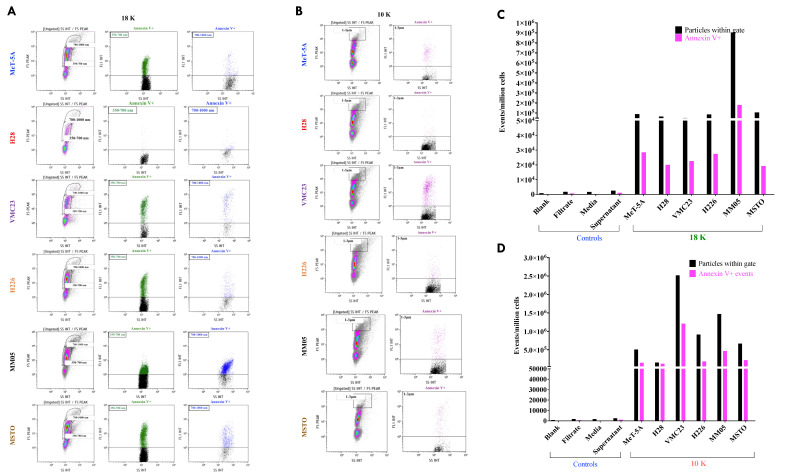

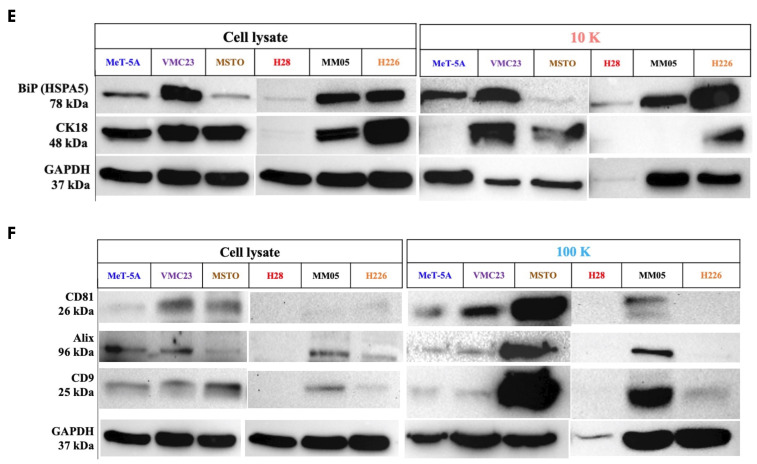
Characterization of EV types. Flow cytometry showing number of gated, phosphatidyl-serine-positive events in (**A**,**C**) 18 K pellets and (**B**,**D**) 10 K EV pellets. (**E**) Western blot with indicated antibodies, showing CK18 and HSPA5 in the 10 K pellets, and (**F**) CD81, CD9, and ALIX in the 100 K pellets. GAPDH was loaded as a control. Original blot see Supplementary File S1.

Figure 3 shows the forward scatter and side scatter cytograms, as well as the gating strategy employed in flow cytometry. All 18 K and 10 K pellets expressed phosphatidylserine (PS) to varying degrees. In the 18 K EVs, MM05 displayed the highest number of Annexin V+ events (Figure 3C), consistent with the NTA results (Figure 2C). MM05 also displayed a concentrated cluster of Annexin V+ events in the large EV gate relative to the other cell lines. All 18 K pellets displayed a higher expression of PS in the smaller-sized gate (350–700 nm) relative to the larger-sized gate (700–1000 nm), indicating a lack of large EVs in the 18 K pellet (Figure 3A). In the 10 K EV samples, all cell-derived 10 K EVs expressed PS (Figure 3B) in the larger-sized gate capturing up to 3 µm sized particles. Moreover, 10 K EVs derived from VMC23 displayed the highest number of Annexin V+ events, and 10 K EVs derived from H28 displayed the lowest number of Annexin V+ events. Four controls were included to account for false-positive events: a blank sample consisting of a buffer with no Annexin V staining; a sample from the filtrate, which is the residual media collected after passing conditioned media through the Amicon^®^ filter units in the final step of EV isolation (Figure 2A); cell culture media directly from the bottle, and the supernatant collected after the final 100 K ultracentrifugation step (EV-depleted media).

Specific markers were used to characterize each EV pellet. In addition, 10 K EVs were characterized with western blotting by testing for the presence of at least cytokeratin 18 (CK18) or heat shock 70 kDa protein 5 (HSPA5), which are suggested markers for large EVs, and 100 K EVs were characterized with western blotting by testing for the presence of at least ALIX or any of the tetraspanins CD9 and CD81. GAPDH was used as an internal control in western blots to verify equivalent amounts of protein (50 µg) for cells and EV samples. The average protein yield was 3.4 mg/mL (range 2.0–4.3 mg/mL) for 10 K pellets, 2.8 mg/mL (range 1.3–3.9 mg/mL) for 18 K pellets, and 1.6 mg/mL (range 0.2–2.9 mg/mL) for 100 K pellets. Western blotting showed that all 10 K pellets expressed at least one large EV marker (Figure 3C). For the 100 K pellets, all but H28 expressed at least one small EV marker (Figure 3D), although the protein amount in the 100 K pellets of the H28 cell line was very low, and a faint band was detected for GAPDH in H28 100 K EVs.

### 3.3. The 10 K, 18 K, and 100 K Pellets Show Distinct Profiles

**Figure 4:**
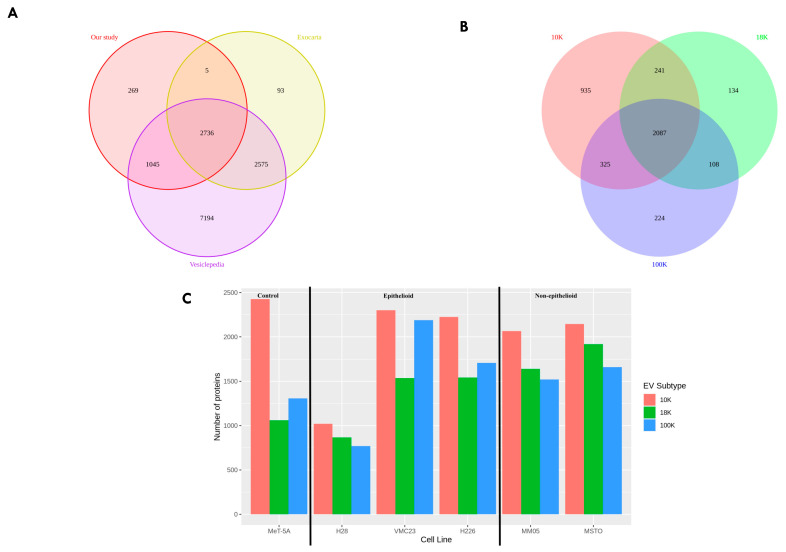
Proteome of EVs. (**A**) Venn diagram showing the overlap between the number of proteins that are enriched in the EVs from this study, with those in the Exocarta and Vesiclepedia databases (ExoCarta Version 5, Release date: 29 July 2015; Vesiclepedia Version 4.1, Release date: 15 August 2018). (**B**) Venn diagram showing the overlap between the number of proteins that are enriched in each of the EV subtypes from this study. (**C**) The number of proteins identified in each of the EV subtypes from each cell line. Data derived from four biological replicates.

**Figure 5:**
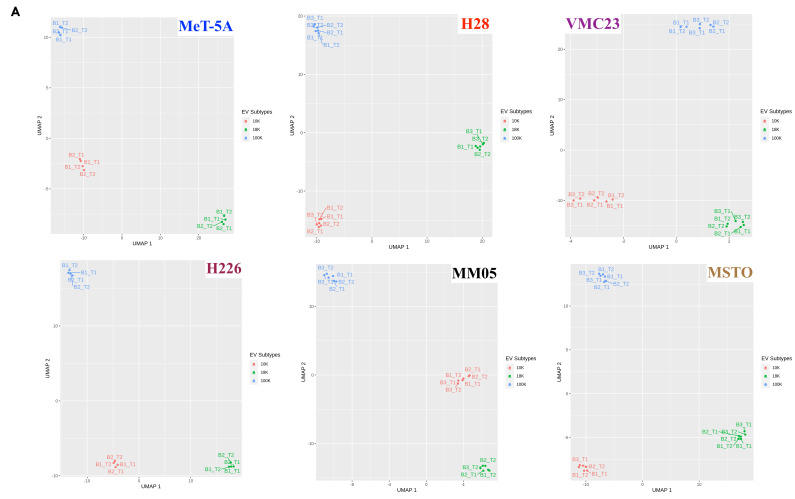

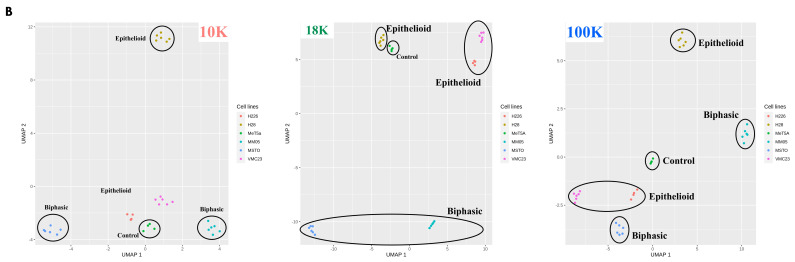
(**A**) UMAP plots showing a very clear separation between the EV subtypes across all cell lines. For each cell line, at least two biological replicates were included and within each biological replicate, two technical replicates were included. (**B**) UMAP plots according to the histological subtype of pleural mesothelioma. B: Biological replication; T: Technical replicate.

A total of 4054 proteins were identified in our study via label-free quantitative proteomic analysis. Of these, 2736 proteins (67%) have been previously reported in the Exocarta and Vesiclepedia (http://microvesicles.org, accessed on 30 August 2021) databases, and 269 proteins were unique to our study (Figure 4A). Between the EV types, 935 proteins were unique to 10 K pellets, 134 proteins were unique to 18 K pellets, and 224 proteins were unique to 100 K pellets (Figure 4B). The total number of proteins extracted from each EV pellet in each cell line is shown in Figure 4C, with H28 having the lowest protein content compared to the other mesothelioma cell lines, consistent with NTA and western blotting results. A 2D visualization of proteomics data after UMAP dimensionality reduction demonstrated a very clear spatial separation between 10 K, 18 K, and 100 K pellets across all cell lines (Figure 5A). The clusters shown for each sample include at least two biological replicates, each with two technical replicates, providing a high level of confidence for the distinction shown between the EV types. We also performed a UMAP plot of the EV types according to the histological subtype (Figure 5B). We found a clear separation only in the 18 K EVs, where all 18 K EVs derived from the epithelioid cell lines (VMC23, H226, and H28) were distinctly separated from the 18 K EVs derived from the non-epithelioid cell lines (MM05 and MSTO). The separation between histological subtypes of pleural mesothelioma is not as clear in the 10 K and 100 K EV groups.

**Figure 6:**
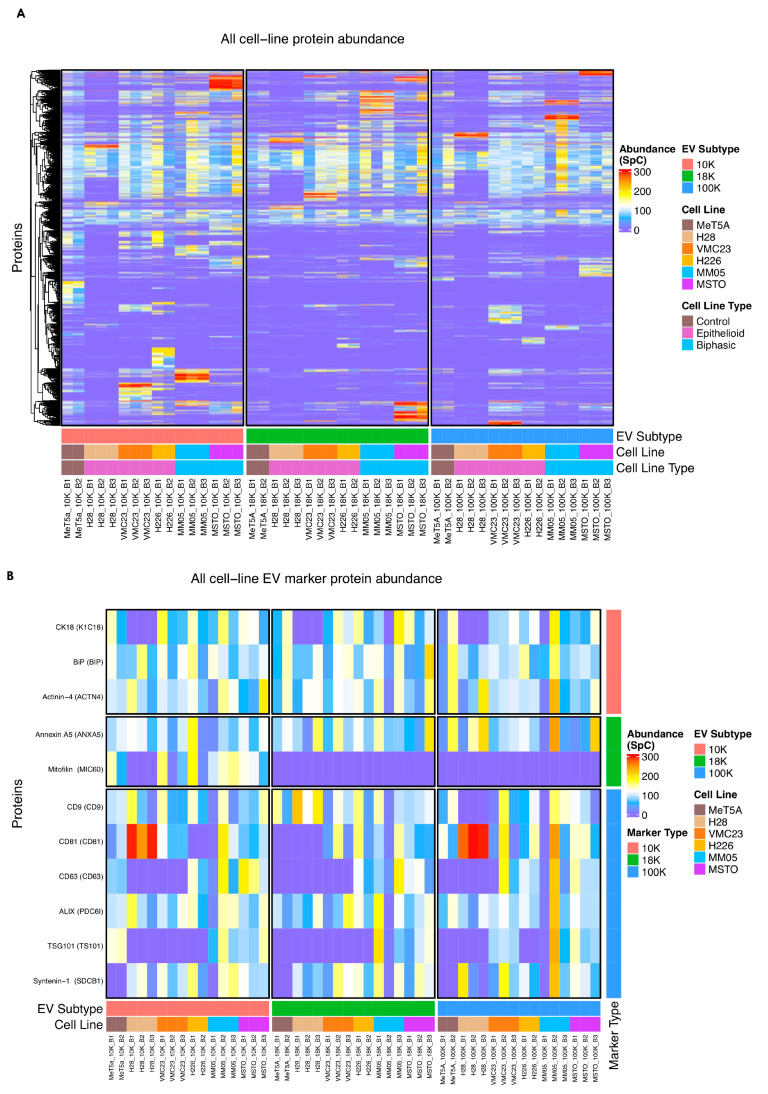
Heatmap representing the differential abundance based on normal spectral count (SpC). (**A**) Heatmap of differentially expressed proteins across all samples, including their biological and technical replicates, showing similarities and differences between each of the EVs derived from pleural mesothelioma cell lines. (**B**) Heatmap focusing on the differential expression of common EV markers across each of the EV subtypes derived from pleural mesothelioma cell lines. Data obtained from four biological replicates. Protein abundance values represent the sum of all unique peptides for a specific protein.

In Figure 6A, a heatmap with hierarchical clustering shows the abundance and protein profiles of all EVs across all cell lines. A cluster of proteins can be observed abundantly across all EV subtypes, indicated by the lighter-colored scales in the upper section of the heat map. There are also small clusters of high abundant proteins that are visible exclusively in a specific subtype of EVs, indicated by the yellow, orange, and red scales. The clusters are more apparent in the 10 K EVs, where multiple clusters can be seen that are absent in the corresponding 18 K and 100 K EV pellets. In Figure 6B, the heatmap focused on markers that have been commonly reported as EV markers. Mitofilin is the only marker that was exclusively differentially expressed in the 10 K EVs.

**Figure 7:**
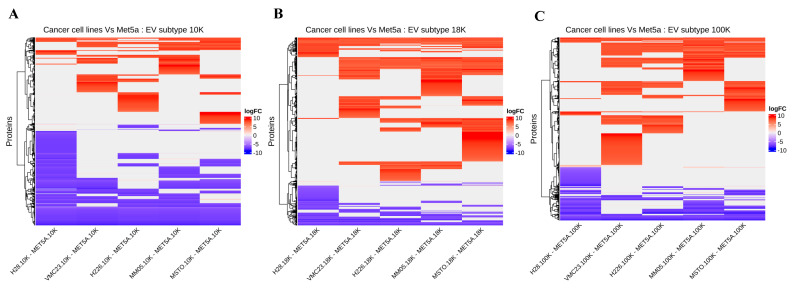
Focused heatmap showing the top overexpressed and underexpressed proteins present in the (**A**) 10 K EV (**B**) 18 K EV, and (**C**) 100 K EV subtypes across all pleural mesothelioma cell lines. Data obtained from four biological replicates.

We also investigated the top five differentially expressed proteins in each EV type compared to the corresponding EV type derived from the control cell line, MeT-5A (Figure 7). In the 10 K EVs, among the top differentially expressed proteins, we found upregulation of leucine-tRNA ligase (*SYLC)*, carbohydrate sulfotransferase 3 (*CHST3*), neogenin (immunoglobulin superfamily DCC subclass member 2) (*NEO1*), aldo-keto reductase family 1 member A1 (*AK1A1*), and the toll-interacting protein (*TOLIP*). We also found downregulation of high-affinity cAMP-specific 3’,5’-cyclic phosphodiesterase 7A (*PDE7A),* nucleolar and coiled-body phosphoprotein 1 (*NOLC1*), microfibrillar-associated protein 1 (*MFAP1*), bisphosphoglycerate mutase (*PMGE*), cystic fibrosis transmembrane conductance regulator (*CFTR*), and pre-mRNA 3’-end-processing factor FIP1 (*FIP1*). In the 18 K EVs, among the top differentially expressed proteins, we found upregulation of asparagine synthetase (*ASNS*), platelet-derived growth factor receptor beta (*PGFRB*), vitamin K-dependent protein S (*PROS*), and ubiquitin-40S ribosomal protein S27a (*RS27*), as well as downregulation of collagen alpha-1 (*COAA1*), protein IWS1 homolog (*IWSI*), gelsolin (*GELS*), and adenylate kinase isoenzyme 1 (*KAD1*). In the 100 K EVs, some of the top differentially expressed proteins include upregulation of aldo-keto reductase family 1 member C2 (*AK1C2*), integrin-linked protein kinase (*ILK*), ATP-dependent DNA helicase Q1 (*RECQ1*), lysyl oxidase homolog 1 (*LOXL1*), and N-alpha-acetyltransferase 15 (*NAA15*), as well as downregulation of multiple inositol polyphosphate phosphatase 1 (*MINP1*), desmoplakin (*DESP*), SAP domain-containing ribonucleoprotein (*SARNP*), and mesencephalic astrocyte-derived neurotrophic factor (*MANF*). These proteins have associations with cancer pathogenesis, as discussed below.

### 3.4. Proteomic Profiles Reveal Specific Biological Processes and Cellular Components Exclusively Enriched in 10 K, 18 K, and 100 K EVs

There were 11 biological processes and 10 enriched cellular components common to all EV pellets (Table 3). Among the biological processes common to all EV types, we observed antigen processing and presentation, neutrophil activation, and degranulation, all of which are known to be involved in inflammation and immune response.

**Figure 8:**
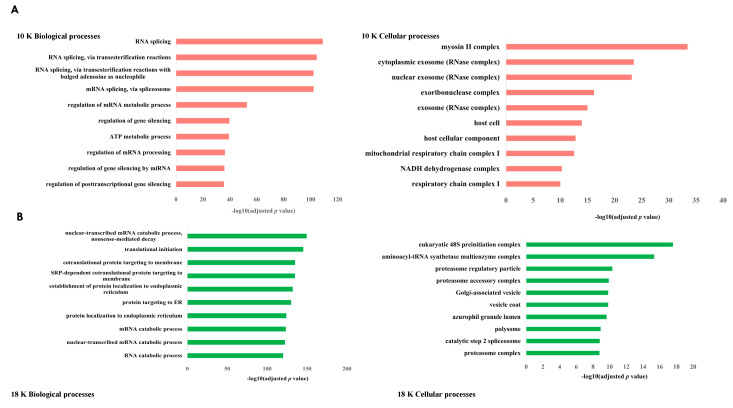

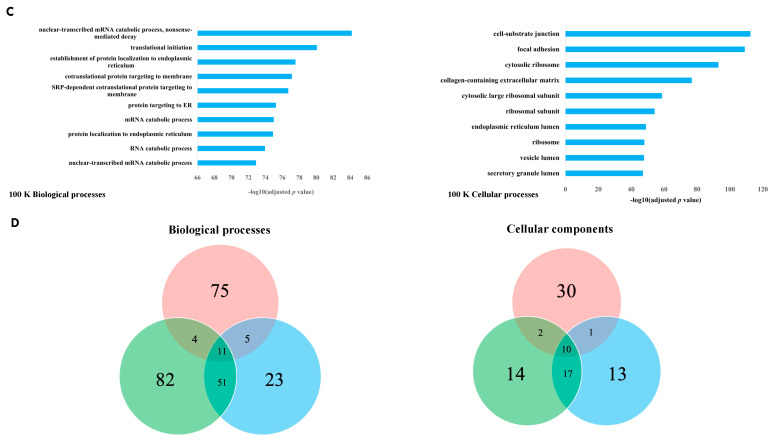
Subcellular location and biological processes of the total identified proteins of each EV subtype derived from mesothelioma cell lines. Top 10 biological processes and enriched cellular components associated with EVs derived from mesothelioma cell lines versus control for (**A**) 10 K EVs, (**B**) 18 K EVs, and (**C**) 100 K EVs. (**D**) Venn diagram showing biological processes and cellular components that are common and unique to each EV pellet from mesothelioma cell lines versus the corresponding control EV pellets. Data obtained from four biological replicates.

The biological processes unique to each EV subtype are provided in Table 4, and the top 10 biological processes and enriched cellular components for each EV subtype are shown in Figure 8.

Four biological processes were common to 10 K and 18 K EVs; five biological processes were common to 10 K and 100 K EVs, while 51 biological processes were common to 18 K and 100 K EVs (Figure 8D), suggesting that 10 K EVs are potentially more distinguished than 18 K and 100 K EVs. In the 10 K EV pellet, unique biological processes included regulation of gene silencing, mRNA export from the nucleus, multi-organism transport, DNA packaging, and metabolic, catabolic, and exocytic processes. In the 18 K EVs, unique biological processes included the Wnt signaling pathway, which is known to play a key role in the development of malignant mesothelioma [28]; other processes also included antigen processing and presentation of exogenous peptide antigens via MHC class I, response to hypoxia, T cell receptor signaling pathway, activation and regulation of innate immune response, RNA transport, and response to interleukin-1, which is an inflammatory cytokine [29]. In the 100 K EVs, some of the unique biological processes identified included protein targeting to membrane, transfer RNA metabolic process, and threonine kinase signaling pathway, which is known to be activated in mesothelioma [30].

There were 17 enriched cellular components common to the 18 K and 100 K EV pellets, but only two enriched cellular components common to the 10 K and 18 K EV pellets and only one enriched cellular component common to the 10 K and 100 K EV pellets (Figure 8D), suggesting again that 10 K EVs are potentially more distinguished than 18 K and 100 K EVs. Among the common enriched cellular components, we found cell-substrate junction, collagen-containing extracellular matrix, and spliceosomal complex enriched in all EV types. Among the cellular components exclusively enriched in 10 K EVs, we identified the mitochondria as a recurring source of enrichment for 10 K EVs. For the 18 K EVs, we found exclusive enrichment in coated membrane, coated vesicle, coated vesicle membrane, membrane coat, and proteasome, all of which are responsible for the degradation of intracellular proteins. Finally for the 100 K EVs, we found exclusive enrichment in ficolin-1-rich granule membrane, ribosome, and specific components of the spliceosome complex, among others listed in Table 5.

### 3.5. Cancer-Associated Proteins in EVs of Pleural Mesothelioma

We then searched our entire proteome database for cancer-associated proteins, or proteins that have been reported in other cancers and associated with tumorigenesis. We found 46 proteins that were present in all the EV pellets from all mesothelioma cell lines and absent in all the EV pellets from the control cell line, MeT-5A (Table 6). Among these included mesothelin (*MSLN*), epidermal growth factor receptor (*EGFR*), fibulin-1 (*FBLN1*), platelet-derived growth factor receptor beta (*PDGFRB*), ras-related protein R-Ras2 (*RRAS2*), protein transport protein (*SC23B* and *SC31A*), tumor necrosis factor receptor superfamily member 10B (*TR10B*), macrophage colony-stimulating factor 1 (*CSF1*), and DNA damage-binding protein 2 (*DDB2*). The full list of proteins and their descriptions are provided in Table 6. Their presence across all EVs derived from mesothelioma cell lines suggests strong biomarker potential for pleural mesothelioma within the cargo of EVs.

**Figure 9:**
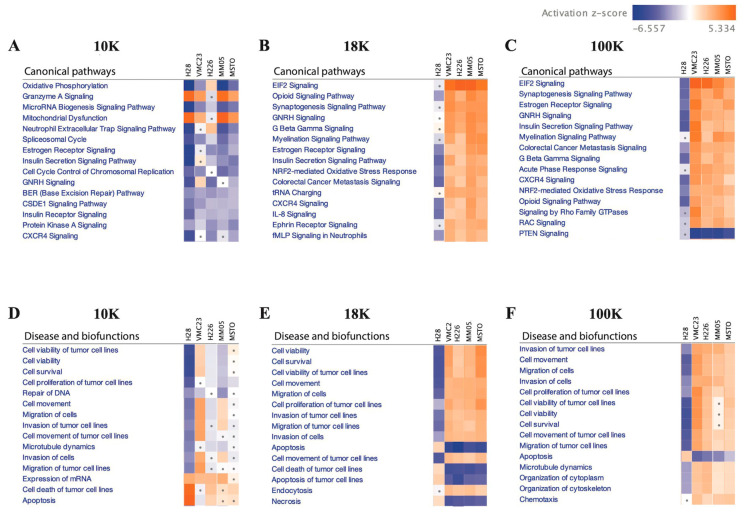
IPA analysis showing the activation or inhibition levels of cancer-related molecular pathways in (**A**) 10 K, (**B**) 18 K, and (**C**) 100 K pellets, and the biological functions in (**D**) 10 K, (**E**) 18 K, and (**F**) 100 K pellets compared to the corresponding MeT-5A control. Data derived from four biological replicates.

Our IPA analysis provided further insights into the activation or inhibition levels of cancer-related molecular pathways and biological functions in EV pellets compared to the corresponding MeT-5A control. Specifically, we found that the 10 K EV pellets showed predominantly decreased oxidative phosphorylation, microRNA biogenesis signaling, and neutrophil extracellular trap signaling pathways (z-score < −2; *p* < 0.05; Figure 9A). Coenzyme A signaling and mitochondrial dysfunctions were activated in the 10 K EV pellets, except for the H226 (z-score > 2; *p* < 0.05). Except for VMC23 and MSTO, all other 10 K EV pellets exhibited decreased cell viability, cell survival, cell proliferation, and DNA repair functions (Figure 9D).

In contrast, the 18 K and 100 K EV pellets, except for H28 (showing decreased or insignificant activity), exhibited significantly increased activation of eukaryotic initiation factor 2 (EIF2) signaling, estrogen receptor signaling, synaptogenesis, and gonadotropin-releasing hormone (GNRH) signaling pathways (Figure 9B,C), as well as increased cell viability, cell survival, cell migration, and cell proliferation, and decreased apoptotic functions (Figure 9E,F). The list of top 15 canonical pathways and biofunctions implicated in the EV groups are presented in Figure 9.

### 3.6. Cancer-Associated Proteins Specific to 10 K, 18 K, and 100 K EVs

**Figure 10:**
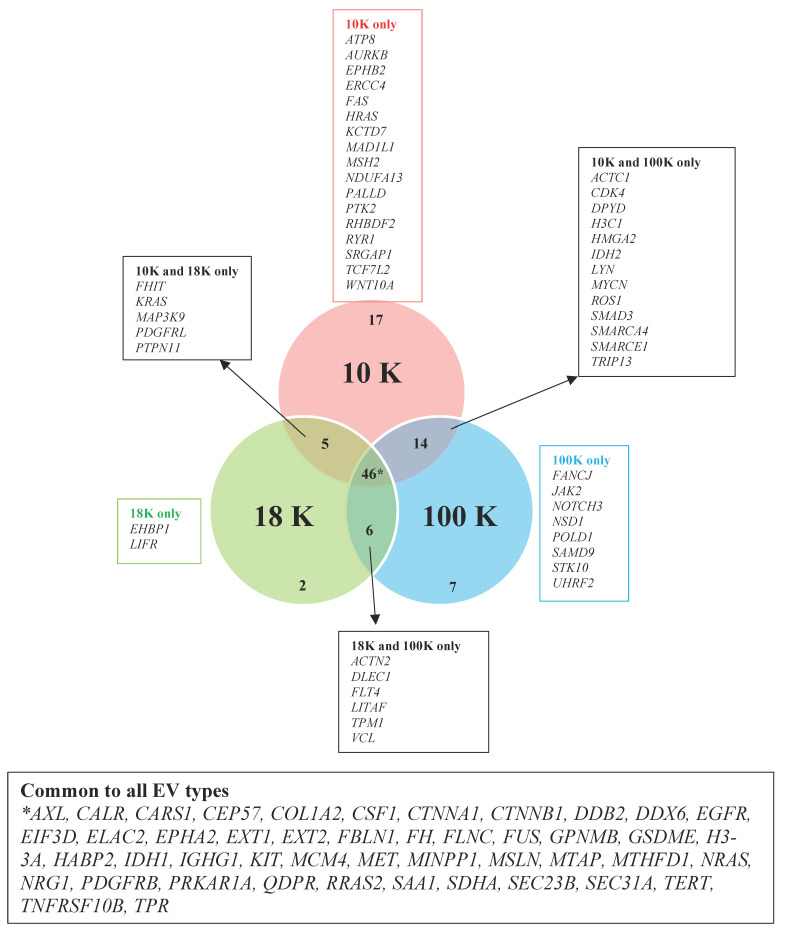
Venn diagram showing cancer-specific proteins identified in this study for each subtype of EVs derived from pleural mesothelioma cell lines. Data derived from four biological replicates.

As shown in Figure 10, there were 17 proteins with links to cancer found exclusively in the 10 K EVs. Among these are NADH-ubiquinone oxidoreductase subunit A13 (*NDUFA13*), which is located in the mitochondrial inner membrane and function as a tumor suppressor [77]; protein Wnt-10a (*WNT10A*), involved in Wnt signaling and has an established role in mesothelioma [78]; transcription factor 7-like 2 (*TCF7L2*), a transcription factor in the Wnt-signaling pathway [79]; mitotic spindle assembly checkpoint protein MAD1 (*MAD1L1)*, a checkpoint gene with its dysfunction associated with chromosol instability [80]; and Fas cell surface death receptor (*FAS*), a member of the tumor necrosis factor-receptor superfamily with a key role in apoptotic signaling pathways and where mutations can prevent the immune system from attacking tumor cells [81]. In the 18 K EVs, two cancer-associated proteins were exclusively found: EH domain-binding protein 1 (*EHBP1*), associated with endocytic trafficking and previously reported in prostate cancer [82,83]; and leukemia inhibitory factor receptor (*LIFR*), involved in the cellular differentiation, proliferation and survival and previously reported in epithelial tumors of the salivary gland [84]. In the 100 K EVs, among the unique cancer-associated proteins, we found notch receptor 3 (*NOTCH3*), which plays a key role in the function and survival of vascular smooth muscle cells [85]; serine/threonine-protein kinase 10 (*STK10*), which functions as a tumor suppressor [86]; janus kinase 2 (*JAK2*), which plays a key role in cytokine and growth factor signaling [87]; and fanconi anemia group J protein (*FANCJ*), which plays an important role in cell cycle checkpoint control [88]. The full list of cancer-associated proteins in each EV type is provided in Figure 10. Details of each protein and their description are provided in Table 7, Table 8 and Table 9.

### 3.7. Mesothelioma Markers Are Localized to Specific EV Types

We then conducted a search of proteins that have established biomarker potential in pleural mesothelioma tumor tissue samples. We found that all these proteins were significantly differentially expressed in specific EV subtypes, suggesting that some of these biomarkers are localized to small or large EVs (Table 10). Among these, pleural mesothelioma-associated proteins include the programmed cell death 1 ligand 2 (PD-L2), which we found to be significantly overexpressed in only the 10 K EVs of VMC23 and MM05; bridging Integrator 1 (Bin1), which we have previously identified as a tumor suppressor and prognostic marker of pleural mesothelioma [89], was significantly under expressed in only the 18 K EVs of all pleural mesothelioma cell lines. Cyclin-dependent kinase inhibitor 2A (CDKN2A) was significantly under expressed in only the 10 K EVs of all cell lines, and cellular tumor antigen p53 (TP53) was found significantly under expressed in only the 10 K EVs of all cell lines (Table 10).

**Figure 11:**
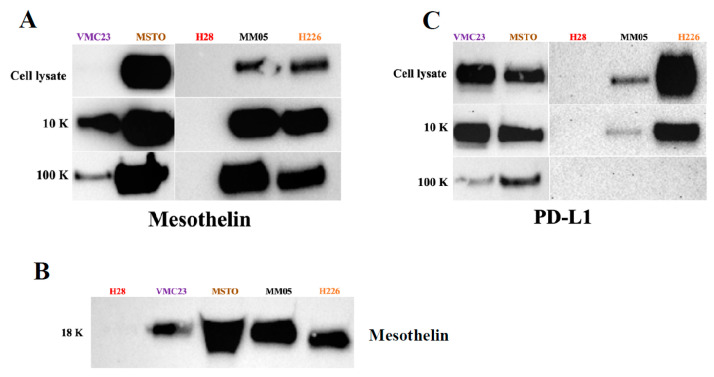
Western blot analysis showing (**A**) the presence of mesothelin in the 10 K and 100 K EVs of mesothelioma cell lines, (**B**) presence of mesothelin in the 18 K EVs of mesothelioma cell lines, and (**C**) presence of PD-L1 in the 10 K EVs of mesothelioma cell lines. Original blot see Supplementary File S1.

We further validated the presence of two key pleural mesothelioma biomarkers, PD-L1 and mesothelin, via western blotting in each EV pellet across all cell lines (Figure 11). PD-L1 was not detected in any of the 18 K EV pellets. PD-L1 was detected in the 10 K EVs of all but H28 mesothelioma cell lines. Interestingly, the enrichment was relatively greater in the 10 K EVs than in the 100 K EVs, consistent with our proteomics results. For MM05 and MSTO, PD-L1 was only detected in the 10 K EVs and not in the 100 K EVs. Mesothelin was detected in all three 10 K, 18 K, and 100 K pellets derived from four of the mesothelioma cells, but not in the EVs derived from H28 cell line, although this was not surprising given the consistently low EVs and protein yield obtained from H28 cells across all experiments.

## 4. Discussion

In this study, we have demonstrated that different subtypes of EVs are released by mesothelioma cancer cells and their cargo can uncover new diagnostic, prognostic, or predictive biomarkers for cancer therapy.

Only two proteomic studies have been published to date on EVs derived from mesothelioma cell lines. However, to our knowledge, there are currently no studies that have investigated EVs derived from the cell lines used in this study. Our study is also the first to investigate different subtypes of EVs from mesothelioma cell lines. In the previous proteomic studies, the focus has been on small EVs, termed exosomes, whereas medium- to large-sized EVs have not yet been studied in mesothelioma cell lines. Greening et al. (2016) [90] conducted quantitative proteomics on pleural mesothelioma-derived exosomes (isolated at 100,000× *g* for 2 h) from four malignant mesothelioma cell lines (J038, LO68, OLD1612, and JU77). They defined a selective mesothelioma oncogenic exosomal signature (mEXOS) consisting of 570 proteins [90]. Of the 570 proteins in mEXOS, 344 proteins (approximately 60%) were identified as complementary DNA (cDNA) and 3 proteins were uncharacterized protein fragments. Of the remaining 224 proteins in mEXOS, 48 proteins were also identified in our proteomics results in this study, most of which were found in all our EV subtypes, with some exceptions (Supplementary Table S1). For example, Annexin A6, which is a calcium-dependent membrane-binding protein that is closely associated with several cancers such as melanoma, epithelial carcinoma, cervical, breast and prostate cancer [91], was not identified in our 100 K EVs, but was found mostly in our 10 K EVs and in only one 18 K EVs derived from the MM05 cell line. This suggests the possibility for membrane-binding proteins to be localized to the larger-sized EVs due to their biogenesis through blebbing from the plasma membrane. Furthermore, ADAMTS12 (which has shown both pro- and anti-tumor roles and associated with immune cells [92]), neuropilin 2 and interleukin-7 (both of which act on immune cells and have shown great potential in cancer immunotherapy [93,94]) were only detected in our 10 K EVs and 100 K EVs, but not in any of our 18 K EVs, suggesting the presence of immune-associated proteins localized to the cargo of large (10 K) and small (100 K) EVs. Furthermore, OASL (potential prognostic biomarker in breast cancer [95]) was only found in one of our 10 K EVs, suggesting that the cargo of 10 EVs contains oncogenic material. Overall, our results complement the protein signature characterized in mEXOS in Greening et al.’s study and uncovers a larger portfolio of proteins of biomarker value for mesothelioma. Our results further show that certain markers are localized to specific subsets of EVs, which can help streamline biomarker identification and tracking for mesothelioma.

Greening et al. also observed 16 mesothelioma-associated proteins in the cargo of their small EVs, the so-called exosomes, of which 14 were also found in the protein cargo of EVs in our study (as indicated in Table 10). These include mesothelin, calretinin, and vimentin. These proteins are already known to be expressed in mesothelioma tumor tissue microenvironment and have been suggested as biomarkers for the differential diagnosis of pleural mesothelioma [96,97,98]. Their presence in EVs across two studies from different mesothelioma cell lines, especially in specific subsets of EVs in our results, opens an exciting pathway to detect and extract these important biomarkers for pleural mesothelioma without invasive tissue biopsy techniques.

In another proteomics study, Hegmans et al. analyzed exosomes derived from two mesothelioma cells (PMR-MM7 and PMRMM8) [99]. They did not find mesothelioma-associated antigens; however, they found 19 proteins that were associated with antigen presentation, including MHC class I molecules. Interestingly, we found antigen presentation via MHC class I exclusively enriched in our 18 K EVs. These results provide insight into the select oncogenic cargo of EV subtypes and support the notion that cancer-specific biomarkers are localized to specific subtypes of EVs, where proteins associated with antigen presentation more likely to be localized to the cargo of the 18 K EVs. In a recent study involving pleural biopsies of 44 mesothelioma patients, Kosari et al. found that multiple antigen processing and presentation gene sets were predictive of overall survival, which could help facilitate patient selection for immunotherapy [100]. Our study suggests that circulating 18 K EVs may offer novel predictive biomarker potential for immunotherapy for mesothelioma patients.

We have uncovered several novel biomarker candidates for pleural mesothelioma by identifying the top upregulated and downregulated proteins that were consistently expressed in each of the EV subtypes derived from all pleural mesothelioma cell lines, compared to the corresponding EVs of the control cell line. Each of these highly over- and under-expressed proteins are involved in cancer pathogenesis and have reported diagnostic, prognostic, or predictive biomarker potential in other cancers from previous reports, suggesting that they have great potential as cancer biomarkers for pleural mesothelioma. In addition, we identified several cancer-associated proteins that were present in all the EV pellets from all five mesothelioma cell lines and absent in all the EV pellets from the control cell line. Among these are proteins such as AXL, which has recently been discovered as a potential target in cancer treatment as it has important signaling functions that drive cancer cell survival, proliferation, migration and invasion and whose aberrant expression has been found in several malignancies including breast cancer, chronic lymphocytic leukemia, and pancreatic cancer [31]; and calreticulin, a protein found in the endoplasmic reticulum that is involved in a spectrum of cellular process including folding of proteins that help stressed and dying cells release co-stimulatory signals to immune cells [32].

Our results further suggest that mitochondrial metabolism may play an important role in pleural mesothelioma tumorigenesis. The mitochondrial enzymes succinate dehydrogenase (*SDHA*), fumarate hydratase (*FH*), and isocitrate dehydrogenase (*IDH1*) were detected in all our EV pellets (10 K, 18 K, and 100 K) derived from all our mesothelioma cell lines but were absent in all EV pellets derived from our control cell line, MeT-5A. It has previously been reported that mitochondrial function is essential for cancer cell viability and that mutations in *SDHA*, *FH*, and *IDH1* [101] can change mitochondrial metabolism and allow cancer cells to adapt to changing environments. They could be potential biomarkers for pleural mesothelioma. Although we detected these enzymes in all EV pellets derived from all our pleural mesothelioma cell lines, the mitochondria were an enriched cellular component exclusively in the 10 K EVs. One hypothesis is that mitochondrial EV cargo may have originated from the large EVs and passed on to other EV types for cellular transport, which warrants further investigation. It has also been suggested that mitochondrial proteins are present in EV cargo even in unstimulated conditions, or that there may be mitochondria-derived vesicles (MDVs), which are small vesicles that carry mitochondrial proteins to other organelles. One study showed that cells selectively package damaged mitochondrial components in MDVs for lysosomal degradation to prevent the release of damaged components or pro-inflammatory content [102]. Although we found mitochondrial proteins in all our EV subtypes, especially in the 10 K EVs, we did not characterize MDVs as this was outside the scope of our study. Our results warrant further investigation into the role of MDVs as a potential EV subtype that may confer biomarker potential.

We also searched for known pleural mesothelioma markers that have been established from tissue samples, to determine if we can detect them in our EVs. Mesothelin and the programmed death ligand-1 (PD-L1) are the most widely reported biomarkers in pleural mesothelioma [103,104]. Mesothelin is a cell surface protein that is expressed normally in mesothelial cells and is elevated in the serum of pleural mesothelioma patients, particularly for epithelioid subtype. Soluble mesothelin has gained regulatory approval for monitoring of patients who are diagnosed with epithelioid or biphasic pleural mesothelioma, albeit in limited situations [97]. It currently lacks the required sensitivity for routine clinical use. PD-L1 is an immune-suppressing receptor that is expressed on tumor cells. It binds to the corresponding PD-1 receptor on T cells to suppress their tumor-killing function. PD-L1 expression in the tumor tissue has been established as marker of poor prognosis in pleural mesothelioma [105]. It is currently being investigated as a predictive biomarker for immunotherapy in multiple clinical trials [106], including in our recent study evaluating the safety and efficacy of pembrolizumab in mesothelioma patients [106]. However, results have so far been equivocal. Recently, Chiarucci et al. (2020) investigated the soluble form of PD-L1 in the sera of pleural mesothelioma patients from the NIBIT-MESO-1 clinical trial [107]. They found elevated levels of soluble PD-L1 in the immunotherapy-treated patients vs. baseline (*p* < 0.001). The study provides the basis to investigate soluble PD-L1 as opposed to tumor PD-L1 expression levels for biomarker potential. This is especially important for pleural mesothelioma due to the lack of tumor availability for robust biomarker studies. Although the origins of the soluble form of PD-L1 is not yet fully understood in pleural mesothelioma, our study alludes to EVs as one possible mechanism. Previously, PD-L1 has only been reported in small EVs, the so-called exosomes, in melanoma [108], non-small cell lung cancer [109], and gastric cancer [110]. Our study is the first to investigate PD-L1 in large EVs, where we found a higher enrichment of PD-L1 expression in our large EV pellets, 10 K, relative to our small EV pellets, 100 K. We did not detect PD-L1 in our 18 K EV pellets. Similarly, we detected mesothelin in our EV pellets. Interestingly, for some of our cell lines, a higher enrichment of mesothelin was detected in our small EVs, 100 K, relative to the 10 K and 18 K EV pellets. Our results give preliminary evidence that PD-L1 and mesothelin are localized to specific subtypes of EVs in pleural mesothelioma, which warrants further investigation. Our results also encourage further investigation of PD-L1 in the cargo of 10 K EVs as a source of biomarker for pleural mesothelioma.

While our large 10 K EVs were mostly enriched with mitochondrial components, interestingly, both our 18 K EVs and 100 K EVs were mostly associated with the endoplasmic reticulum (ER). Some of the top biological processes associated with the 18 K EVs and 100 K EVs were protein targeting to the ER, protein localization to the ER, and SRP-dependent co-translational protein targeting to the membrane, which is a protein that binds to the ER [111]. The ER lumen was also one of the top enriched components in the 18 K EVs and 100 K EVs. It is known that protein handling and folding in the ER are critical processes for cell function and survival. In the tumor microenvironment, oncogenic and metabolic abnormalities can create aberrant activation of ER stress signals that could in turn activate aberrate signaling pathways for tumor growth [112]. ER stress is therefore an important characteristic of the tumor microenvironment. Some studies suggest that the modulation of ER stress can make aggressive tumors sensitive to cytotoxic drugs and immunotherapies [112]; however, this needs to be investigated further. We observed that there are many overlaps in the biological processes and cellular components between 18 K EVs and 100 K EVs; however, 10 K EVs in comparison do not share many common biological processes or enriched cellular components to either the 18 K EVs or the 100 K EVs. Our results suggest that EV isolation at 10 K results in the isolation of more distinct EV subtypes that carry unique oncogenic cargo and should be investigated more in future EV studies.

Additionally, IPA analysis of the data revealed that 10 K EV pellets exhibit a distinct proteomic profile, characterized by decreased activities of oxidative phosphorylation, microRNA biogenesis signaling, and neutrophil extracellular trap signaling pathways. These pathways are known to interplay and promote tumor growth, progression, and metastasis when activated [113,114]. The observed reduction in protein activation in these pathways may account for the decreased cell viability, cell survival, cell proliferation, and DNA repair as seen in the 10 K EVs. Further investigations are required to verify these results; however, one possible explanation is that the exosomes may contain more carcinogenic materials and are primarily present in fractions isolated at speeds greater than 10 K [115]. Nonetheless, we did report in this study that 10 AK EVs still carries potential protein biomarkers that warrants further investigation.

Conversely, a majority of the 18 K and 100 K EV pellets exhibited increased activation of various pathways, including the EIF2 signaling and NRF2-mediated oxidative stress response pathways. Our findings align with prior research that demonstrated that increased expression and activity of EIF2 [116] can promote the growth of mesothelioma. NRF2 has also been reported to play a critical role in the proliferation of MSTO-211H cell lines by elevating intracellular ROS levels [117]. These results are consistent with our observation that hallmark cancer features, such as cell viability, cell migration, and cell proliferation, are enhanced, while apoptotic activity is decreased in the 18 K and 100 K EVs. Overall, our results suggest that EV isolation at 10 K results in the isolation of more distinct EV subtypes that carry limited but unique oncogenic cargo and should be investigated more in future EV studies.

There are currently limited reports in the literature on different EV subtypes and their role in cancer. Our study encourages further research especially in 10 K EVs, as we have shown important oncogenic cargo to be present exclusively in 10 K EVs derived from pleural mesothelioma cells. In other cancers, there is a dominant focus on small EVs, the so-called exosomes, in the EV literature, with relatively limited reports on larger-sized EVs. Perhaps one of the barriers to studying sub-populations of EVs is the lack of unique markers that can characterize and distinguish them from each other. Crescitelli et al. (2020) investigated subpopulations of EVs derived from metastatic melanoma tissue by isolating EVs at 16,500× *g* for 20 min and then at 118,000× *g* for 2.5 h to isolate large and small EVs, respectively [16]. They found mitofilin (a mitochondrial inner membrane protein) exclusively enriched in the large EVs. Similarly in our results, mitofilin was found only in the 10 K EVs derived from all but H28 cell lines, and absent across the 18 K and 100 K EVs derived from all cell lines, suggesting that it could potentially be a unique marker of 10 K EVs. We also detected HSPA5 (BiP) only in our 10 K EVs, whereas CK18 (previously reported as a marker of large oncosomes [21]) was detected in some of our 10 K EVs and in some of our 100 K EVs. Our results suggest that mitofilin and BiP may be better markers of 10 K EVs. We further characterized 18 K and 10 K EVs using flow cytometry with the EVs stained with Annexin V, which has a high affinity to PS. PS is a phospholipid that is located on the inner membrane of cells or EVs and is translocated to the outer membrane in response to an event or stimuli, such as a vesicular event [118]. EVs, which form from blebbing or shedding of the plasma membrane, are typically characterized by the presence of PS on their outer membrane [119], which prompted us to further characterize 18 K and 10 K EVs using this technique.

For small EVs, tetraspanins including CD9, CD81, and CD63 have been commonly reported as markers of small EVs, or so-called exosomes. However, tetraspanins have been shown to induce plasma membrane curvature, making them present in shedding vesicles [120]. This may explain why we observed enrichment of CD9 in our 10 K EVs (Supplementary Figure S1). This is consistent with the results from Javadi et al. (2021), who conducted a proteomics study of EV subpopulations derived from pleural mesothelioma pleural effusion samples [121] and found enrichment of cell surface tetraspanins CD9 in their large EV pellet (isolated at 10,000× *g* for 10 min and referred to as microvesicles), and enrichment of CD81 in their small EV pellet (isolated 100,000× *g* for 90 min and referred to as exosomes). We also observed enrichment of CD81 in our 100 K EVs, suggesting that CD81 may be a better marker of 100 K EVs. It is noted that currently, there is no single marker that can uniquely identify EVs [122,123]. It should also be noted that EVs can also contain proteins that are considered pan-EV markers, i.e., they are common for most EV subtypes [123], and our results suggest that most of the commonly used EV markers could possibly be pan-EV markers. Our results also showed that the enriched biological processes and cellular processes are distinct in the 10 K EVs compared to 18 K and 100 K EV subtypes. Furthermore, our NTA and TEM results demonstrate that there is substantial overlap in the size of EVs across the three subtypes. This suggests that biogenesis pathway may be a better way of classifying EV subtypes, rather than by the size and the currently available makers.

The main strength of our study is that we performed a pooled analysis of potential biomarkers for pleural mesothelioma from five mesothelioma cell lines of different histological subtypes and an immortalized control cell line, each with at least two biological replicates, and two technical replicates within every biological sample. To our knowledge, this is the largest proteomics study conducted on EVs derived from mesothelioma cell lines to date. This study took a discovery approach to uncover the proteomic profile of pleural mesothelioma and identify a comprehensive list of markers that have cancer biomarker potential. We have revealed several oncogenic targets for mesothelioma, and while we have argued that each of the enriched proteins have associations with cancer pathogenesis from other reports, a follow-up study is strongly encouraged to validate the biomarker potential of each of the key targets discovered in this study for mesothelioma.

The other key strength of our study is that we studied potential biomarkers within different EV populations, which enabled us to reveal multiple targets and uncover the distinct roles of three EV subtypes. Our study opens a pathway of accessing key proteins of biomarker potential more directly from the oncogenic cargo of specific EV subtypes, rather than from a pooled pellet of EVs, or via invasive tissue extraction techniques. This would be a significant step towards improving biomarker access for pleural mesothelioma. We acknowledge that there is vast heterogeneity in the size and populations of EV subtypes that are still under research and not fully understood. In this study, we show that at least three EV subtypes can be isolated using different centrifugal forces, i.e., 10,000× *g*, 18,000× *g* and 100,000× *g* to isolate 10 K, 18 K, and 100 K EVs, respectively. These three subtypes showed many overlapping similarities, as well as many unique properties.

There were also limitations to our study that should be considered for future studies. Firstly, it is now recognized that the methods of EV isolation have limited association with the method of EV formation, and EV isolation protocols typically adopt a purification step. We chose not to use density gradient separation techniques for isolating 10 K and 18 K EVs but chose to use a sucrose cushion for isolating the 100 K EVs. While gradient separation techniques offer higher specificity and are intended to remove as many non-vesicular components and debris as possible, it is acknowledged that this technique is still under research, has the risk of producing low EV yield or recovery, and has not been optimized for 10 K and 18 K EVs. Therefore, we chose to isolate 18 K and 10 K using the differential centrifugation method, which was originally the primary EV isolation technique reported in the literature. This was to ensure that we capture a higher EV yield, at the risk of potentially compromising the purity of the EV pellets retrieved [122]. Our isolation technique for 10 K and 18 K may have caused some of the overlap in the presence of surface EV markers we observed in both the large and small EV pellets. We were also limited with the NTA technology for the particle size distribution because large particles are less likely to be reliably detected by the NTA. Therefore, we were limited to a particle size of 1 μm in our current study and relied on flow cytometry with a specific membrane marker and gating strategy to detect larger-sized EVs. Further optimization of isolation techniques is needed for future EV studies. Despite this, our results showed a predominantly large EV population in our 10 K pellets, a relatively smaller EV population in our 18 K pellets, and small EV populations in our 100 K pellets. Furthermore, our UMAP plots show a clear separation between the three EV subtypes, indicating major differences in the cargo between 10 K, 18 K, and 100 K EVs. Furthermore, MeT-5A may not have been a robust control cell line for mesothelioma. MeT-5A is an immortalized cell line that has been transfected with the simian virus 40 (SV40), which has been proposed as a risk factor for mesothelioma [124]. Although a direct causal link between SV40 and human mesothelioma has not been established, future experiments should consider the use of a mesothelial control cell line without any confounding risk factors.

Given the lack of standardized nomenclature and isolation techniques for EVs, we avoided assigning naming conventions to our EV pellets. We were also limited with the NTA technology for the particle size distribution because large particles are less likely to be reliably detected using the NTA. Therefore, we were limited to a particle size of 1 µm in our current study and relied on flow cytometry with a specific membrane marker and gating strategy to detect larger-sized EVs. Improvements in EV technologies are needed to detect particles larger than 1 µm.

We note that the morphology of our cell lines did not represent the typical characteristics of epithelioid and non-epithelioid subtypes from pleural mesothelioma tumor histology. Epithelioid cells are typically round or polygonal, while sarcomatoid cells are spindle-like, and biphasic cells are a mixture of both. These characteristics have been observed in tissues from patient samples, whereas cells in culture may not be true representations of the histological subtypes of pleural mesothelioma as they are not in their ideal condition of cell growth. Therefore, we were unable to establish clear distinctions between epithelioid and non-epithelioid subtypes in our current study; however, our results encourage further research to investigate biomarkers unique to each subtype, perhaps from patient plasma or pleural effusion samples.

## 5. Conclusions

In summary, we have provided a comprehensive proteomic database of cancer associated proteins in EVs that can offer new targets for future biomarker studies in pleural mesothelioma. We have also demonstrated that different subtypes of EVs can be isolated, namely 10 K, 18 K, and 100 K, each carrying oncogenic cargo with biomarker potential for pleural mesothelioma. These distinct EV populations can help narrow the molecular targets for diagnostic, prognostic, and predictive biomarker studies. One of our major findings is the oncogenic cargo detected in our 10 K EVs, particularly the presence of established pleural mesothelioma biomarkers such as PD-L1 in our 10 K EVs, which warrants further biomarker validation studies. There is currently a predominant focus on exosomes or small EVs, and our study provides the basis to investigate other EV populations for biomarker studies in pleural mesothelioma.

## Figures and Tables

**Table 1:** List of cell lines used in this study.

Cell Line	Histological Subtype	Source
MeT-5A	Immortalized human mesothelial cell line	ATCC, Rockville
H28	Epithelioid	ATCC, Rockville
VMC23	Epithelioid	Medical University of Vienna, Austria
H226	Epithelioid	ATCC, Rockville
MM05	Biphasic (non-epithelioid)	The Prince Charles Hospital, Brisbane
MSTO-211H	Biphasic (non-epithelioid)	ATCC, Rockville

ATCC: American Type Culture Collection (Manassas, VA, USA).

**Table 2:** List of primary antibodies used in this study.

Primary Antibody	Clone	Source	Dilution
Anti-CD9	D3H4P Rabbit mAb	Cell Signaling Technology	1:1000
Anti-CD81	D3N2D Rabbit mAb	Cell Signaling Technology	1:1000
Anti-TSG101	5B7 Mouse mAb	Merck	1:1000
Anti-ALIX	3A9 Mouse mAb	Cell Signaling Technology	1:1000
Anti-mitofilin	EPR8749 Rabbit mAb	Abcam	1:1000
Anti-alpha-Actinin 4	D7U5A) Rabbit mAb	Cell Signaling Technology	1:1000
Anti- Cytokeratin 18	DA-7 Mouse mAb	Biolegend	1:1000
Anti-BiP (HSPA5)	C50B12 Rabbit mAb	Cell Signaling Technology	1:1000
Anti-Mesothelin	D9R5G XP(R) Rabbit mAb	Cell Signaling Technology	1:1000
Anti-PD-L1	E1L3N(R) XP(R) Rabbit mAb	Cell Signaling Technology	1:1000
Anti-BIN1	14647-1-A Rabbit pAb	Proteintech Group	1:1000
Anti-GAPDH	D16H11) XP(R) Rabbit mAb	Cell Signaling Technology	1:1000

**Table 3:** Biological processes and cellular processes common to all EV pellets across all mesothelioma cell lines.

Biological Processes	Cellular Components
Cell-substrate adhesion	Lysosomal lumen
Neutrophil degranulation	Azurophil granule
Antigen processing and presentation	Cell-substrate junction
Nuclear transport	Collagen-containing extracellular matrix
Neutrophil activation	Endoplasmic reticulum lumen
Neutrophil activation involved in immune response	Focal adhesion
Neutrophil-mediated immunity	Lamellipodium
Regulation of translation	Primary lysosome
Regulation of cellular amide metabolic process	Spliceosomal complex
Nucleocytoplasmic transport	Vacuolar lumen
Golgi vesicle transport	

**Table 4:** Biological processes exclusive to each EV pellet.

10 K	18 K	100 K
Regulation of mRNA metabolic process	Regulation of cellular amino acid metabolic process	Viral gene expression
Regulation of gene silencing	Wnt signaling pathway, planar cell polarity pathway	Protein targeting to membrane
ATP metabolic process	Cell growth	Viral transcription
Regulation of mRNA processing	Regulation of transcription from RNA polymerase II promoter in response to hypoxia	Establishment of protein localization to membrane
Regulation of gene silencing by miRNA	Regulation of establishment of planar polarity	Protein targeting
Regulation of posttranscriptional gene silencing	Biological process involved in interaction with host	Ribonucleoprotein complex biogenesis
Regulation of gene silencing by RNA	Antigen processing and presentation of exogenous peptide antigen via MHC class I	Platelet degranulation
mRNA export from nucleus	Morphogenesis of a polarized epithelium	Negative regulation of endopeptidase activity
mRNA-containing ribonucleoprotein complex export from nucleus	Establishment of planar polarity	Small molecule catabolic process
Nuclear export	Establishment of tissue polarity	Nucleotide-excision repair, DNA damage recognition
Energy derivation by oxidation of organic compounds	Antigen processing and presentation of exogenous peptide antigen via MHC class I, TAP-dependent	Homotypic cell–cell adhesion
Cellular respiration	Non-canonical Wnt signaling pathway	Transmembrane receptor protein serine/threonine kinase signaling pathway
RNA 3’-end processing	Cellular response to oxygen levels	Multi-multicellular organism process
Regulation of mRNA stability	Interleukin-1-mediated signaling pathway	Regulation of actin filament-based process
Regulation of RNA stability	Regulation of cellular amine metabolic process	Lung development
Electron transport chain	Response to oxygen levels	Respiratory tube development
Purine-containing compound metabolic process	Cellular response to decreased oxygen levels	Respiratory system development
Regulation of mRNA catabolic process	Cellular response to hypoxia	Negative regulation of hydrolase activity
Purine nucleotide metabolic process	Response to hypoxia	Nucleobase biosynthetic process
Ribose phosphate metabolic process	Movement in host environment	Positive regulation of cell morphogenesis involved in differentiation
Respiratory electron transport chain	Response to decreased oxygen levels	TRNA metabolic process
Nucleoside monophosphate metabolic process	Cellular response to interleukin-1	Nucleotide-sugar biosynthetic process
Post-Golgi vesicle-mediated transport	Regulation of DNA-templated transcription in response to stress	
Endomembrane system organization	Hematopoietic stem cell differentiation	
Exonucleolytic catabolism of deadenylated mRNA	T cell receptor signaling pathway	
Response to heat	Activation of innate immune response	
Ribonucleotide metabolic process	SCF-dependent proteasomal ubiquitin-dependent protein catabolic process	
Transport of virus	Response to interleukin-1	
Multi-organism localization	Amine metabolic process	
Multi-organism transport	Positive regulation of neurogenesis	
Nucleoside phosphate biosynthetic process	Regulation of transcription from RNA polymerase II promoter in response to stress	
Nuclear-transcribed mRNA catabolic process, exonucleolytic	Positive regulation of cell growth	
Protein-DNA complex subunit organization	Negative regulation of G2/M transition of mitotic cell cycle	
Nucleotide biosynthetic process	Stimulatory C-type lectin receptor signaling pathway	
Endosomal transport	Regulation of protein-containing complex assembly	
Purine ribonucleotide metabolic process	Regulation of cell development	
Response to temperature stimulus	Alpha-amino acid metabolic process	
DNA conformation change	Glycoside metabolic process	
Chromatin assembly or disassembly	Innate immune response activating cell surface receptor signaling pathway	
Mitochondrial electron transport, NADH to ubiquinone	Negative regulation of cell cycle G2/M phase transition	
Regulation of cellular response to heat	Regulation of animal organ morphogenesis	
Megakaryocyte differentiation	Positive regulation of nervous system development	
Intracellular transport of virus	Cellular ketone metabolic process	
Mitotic nuclear division	Negative regulation of Wnt signaling pathway	
Nucleoside monophosphate biosynthetic process	Regulation of G2/M transition of mitotic cell cycle	
DNA packaging	Entry into host	
Regulation of intracellular transport	Positive regulation of innate immune response	
Ribonucleoside monophosphate metabolic process	Sulfur compound biosynthetic process	
Protein localization to mitochondrion	Regulation of cell cycle G2/M phase transition	
Nucleotide-excision repair	Vesicle budding from membrane	
Protein localization to cell periphery	Positive regulation of growth	
Cellular response to heat	G2/M transition of mitotic cell cycle	
Cellular metabolic compound salvage	Proteasome-mediated ubiquitin-dependent protein catabolic process	
Carbohydrate catabolic process	Alpha-amino acid biosynthetic process	
Mitotic spindle organization	Cell cycle G2/M phase transition	
Exocytic process	Adherens junction organization	
Purine nucleoside monophosphate metabolic process	Negative regulation of supramolecular fiber organization	
Protein peptidyl-prolyl isomerization	Positive regulation of defense response	
Peptidyl-proline modification	Regulation of neurogenesis	
Regulation of nucleocytoplasmic transport	Regulation of chemotaxis	
Response to peptide hormone	Aspartate family amino acid metabolic process	
Ribonucleoside diphosphate metabolic process	Proteasomal protein catabolic process	
Purine ribonucleoside monophosphate metabolic process	Axonogenesis	
Protein localization to plasma membrane	Cellular amino acid biosynthetic process	
Purine nucleoside diphosphate metabolic process	Nucleic acid transport	
Purine ribonucleoside diphosphate metabolic process	RNA transport	
Carbohydrate derivative catabolic process	Establishment of RNA localization	
Nucleoside diphosphate metabolic process	Positive regulation of axonogenesis	
Nucleotide phosphorylation	Gland morphogenesis	
Modulation by virus of host process	Regulation of cell-substrate adhesion	
Regulation of biological process involved in symbiotic interaction	RNA localization	
ADP metabolic process	Negative regulation of ubiquitin-dependent protein catabolic process	
Organelle fission	L-serine metabolic process	
Regulation of viral process	Regulation of RNA binding	
Regulation of DNA metabolic process	Positive regulation of translation	
	Regulation of nervous system development	
	COPII-coated vesicle budding	
	Positive regulation of cellular amide metabolic process	
	Serine family amino acid biosynthetic process	
	Serine family amino acid metabolic process	
	Nucleobase-containing compound transport	
	Regulation of substrate adhesion-dependent cell spreading	

**Table 5:** Cellular components unique to each EV type derived from mesothelioma cell lines.

10 K	18 K	100 K
Cytoplasmic exosome (RNase complex)	Adherens junction	Chromosome, telomeric region
Exoribonuclease complex	Coated membrane	Cytosolic large ribosomal subunit
Exosome (RNase complex)	Coated vesicle	Ficolin-1-rich granule membrane
Filopodium	Coated vesicle membrane	Lamellipodium membrane
Host cell	Endopeptidase complex	Large ribosomal subunit
Host cellular component	Eukaryotic 48S preinitiation complex	Platelet alpha granule
Inner mitochondrial membrane protein complex	Golgi-associated vesicle	Platelet alpha granule lumen
Microtubule associated complex	Membrane coat	Precatalytic spliceosome
Midbody	Peptidase complex	Ribosome
Mitochondrial inner membrane	Polysome	Small nuclear ribonucleoprotein complex
Mitochondrial matrix	Proteasome accessory complex	Spliceosomal snRNP complex
Mitochondrial protein-containing complex	Proteasome complex	U2-type precatalytic spliceosome
Mitochondrial respirasome	Proteasome regulatory particle	U2-type spliceosomal complex
Mitochondrial respiratory chain complex I		
Myosin II complex		
NADH dehydrogenase complex		
Nuclear chromosome		
Nuclear exosome (RNase complex)		
Nuclear pore		
Oxidoreductase complex		
Phagocytic vesicle		
Protein-DNA complex		
Respirasome		
Respiratory chain complex		
Respiratory chain complex I		
Secretory granule membrane		
Spindle		
Spindle pole		
Vacuolar membrane		
Z disc		

**Table 6:** Details of proteins that are present in all the EV pellets from all five mesothelioma cell lines and absent in all of the EV pellets from the control cell line (MeT-5A).

*Gene*	Protein Name	Function
*AXL*	Tyrosine-protein kinase receptor	Associated with tumor cell growth, metastasis, invasion, epithelial-mesenchymal transition, angiogenesis, drug resistance, immune regulation, and stem cell maintenance [31].
*CALR*	Calreticulin	Found in the endoplasmic reticulum and helps in correct protein folding [32].
*CARS1*	Cysteinyl-tRNA synthetase 1	Plays an important role in protein synthesis [33] and inhibits non-apoptotic cell death [34].
*CEP57*	Centrosomal protein of 57	Involved in intracellular transport processes and found to be overexpressed in prostate cancer [35].
*COL1A2*	Collagen alpha-2(I) chain	Found to be downregulated in melanoma and bladder cancer. May have prognostic biomarker value in hypopharyngeal squamous cell carcinoma [36].
*CSF1*	Macrophage colony-stimulating factor 1	A critical growth factor for macrophage development. Associated with poor survival in various tumor types [37].
*CTNNA1*	Catenin alpha-1	Involved in cell–cell adhesion and nuclear signaling [38].
*CTNNB1*	Catenin beta-1	
*DDB2*	DNA damage-binding protein 2	Plays a key role in mediating apoptosis following DNA damage [39].
*DDX6*	Probable ATP-dependent RNA helicase DDX6	Involved in most cellular processes that require manipulation of RNA structure. Implicated in cellular proliferation and neoplastic transformation [40].
*EGFR*	Epidermal growth factor receptor	Involved in cell signaling pathways that control cell division and survival. Often over-expressed in human carcinomas [41].
*EIF3D*	Eukaryotic translation initiation factor 3 subunit D	Known to regulate the growth of several types of human cancer cells. Associated with different pathological conditions, including cancer [42].
*ELAC2*	Zinc phosphodiesterase ELAC protein 2	Prostate cancer susceptibility gene [43].
*EPHA2*	Ephrin type-A receptor 2	A class of receptor tyrosine kinases that is highly produced in tumor tissues, while found at relatively low levels in most normal adult tissues [44].
*EXT1*	Exostosin-1	EXT1 is a key regulator of endoplasmic reticulum morphology and dynamics [45]. EXT1 and EXT2 reported as target antigens in secondary (autoimmune) membranous nephropathy [46].
*EXT2*	Exostosin-2	
*FBLN1*	Fibulin-1	A fibrinogen-binding blood protein and a component of many extracellular matrices including those of blood vessels [47].
*FH*	Fumarate hydratase, mitochondrial	An enzyme found in both the cytoplasm and mitochondria can function as a tumor suppressor [48].
*FLNC*	Filamin-C	A member of the actin binding protein family, which is expressed in the cardiac and skeletal muscles [49].
*FUS*	RNA-binding protein FUS	A nucleoprotein that functions in DNA and RNA metabolism, including DNA repair, and the regulation of transcription, RNA splicing, and export to the cytoplasm [50].
*GPNMB*	Transmembrane glycoprotein NMB	Associated with cancer progression and metastasis [51].
*GSDME*	Gasdermin-E	Acts as a tumor suppressor in melanoma, breast cancer, and colorectal cancer [52].
*H3-3A*	Histone H3.3	Expressed throughout the cell cycle, as well as in quiescent cells. Recently found to be mutated at high frequency in several specific cancer types including pediatric high-grade glioblastoma, chondroblastoma, and giant cell tumors of the bone [53].
*HABP2*	Hyaluronan-binding protein 2	Upregulated in non-small cell lung cancer [54].
*IDH1*	Isocitrate dehydrogenase	An essential enzyme for cellular respiration. Mutations in IDH1 are prevalent in several cancers including glioma, acute myeloid leukemia, cholangiocarcinoma, and chondrosarcoma [55].
*IGHG1*	Immunoglobulin heavy constant gamma 1	Associated with immune evasion mechanisms in pancreatic cancer [56] and prostate cancer [57,58].
*KIT*	Mast/stem cell growth factor receptor	A member of the tyrosine kinase family of growth factor receptors that is deregulated in diseases including cancer [59].
*MCM4*	DNA replication licensing factor	Licensing proteins are inappropriately expressed at an early stage of tumorigenesis [60].
*MET*	Hepatocyte growth factor receptor	HGF/Met signaling contributes to oncogenesis and tumor progression in several cancers and promotes aggressive cellular invasiveness [61].
*MINPP1*	Multiple inositol polyphosphate phosphatase 1	Play key signaling roles in diverse cellular functions, including calcium homeostasis, cell survival, and death [62].
*MSLN*	Mesothelin (CAK1 antigen)	An established biomarker that is overexpressed in pleural mesothelioma [63].
*MTAP*	S-methyl-5’-thioadenosine phosphorylase	Deficiency supports melanoma development and progression. Many tumors lack expression of MTAP [64].
*MTHFD1*	C-1-tetrahydrofolate synthase, cytoplasmic	An enzyme in the cytoplasm that has been associated with increased risk for a number of folate-related pathologies, including cancer, although a clear link has not been established [65].
*NRAS*	GTPase NRas	Detected in various cancer cell line EVs and in urine EVs. Associated with cetuximab- and panitumumab-targeted cancer therapies [14].
*NRG1*	Pro-neuregulin-1, membrane-bound isoform	A member of the epidermal growth factor family of receptor tyrosine kinase protein ligands and involved in the activation of proliferation, survival, and differentiation of cells in many tissue types [14].
*PDGFRB*	Platelet-derived growth factor receptor beta	Belongs to the receptor tyrosine kinase family of proteins. Platelet-derived growth factor signaling has associated with cancer [66].
*PRKAR1A*	cAMP-dependent protein kinase type I-alpha regulatory subunit	Found to be up-regulated in a series of cell lines and human neoplasms, suggesting involvement in tumorigenesis [67].
*QDPR*	Dihydropteridine reductase	Related to oxidative stress and associated with activation of mTOR signaling pathway [68].
*RRAS2*	Ras-related protein R-Ras2	Part of the R-Ras GTPase subfamily that is involved in cell signaling and in which mutations have been found to be oncogenic drivers in many cancers [69].
*SAA1*	Serum amyloid A-1 protein	A multifunctional protein that has been reported to upregulate the expression of various inflammatory mediators such as cell adhesion molecules, cytokines, chemokines, matrix-degrading proteases, reactive oxygen species and pro-angiogenic molecules in several cell types including leukocytes, fibroblasts, and endothelial cells [70].
*SDHA*	Succinate dehydrogenase	An enzyme complex found to be a predisposing factor in hereditary cancers [71]. It is the only enzyme that participates in both the citric acid cycle and the electron transport chain [72].
*SEC23B*	Protein transport protein	Promotes the survival of cancer cells [73].
*SEC31A*	Protein transport protein	
*TERT*	Telomerase reverse transcriptase	Plays a major role in the replication and self-renewal of cancer [74].
*TNFRSF10B*	Tumor necrosis factor receptor	Widely accepted as a tumor-suppressive cytokine via its ubiquitous receptor TNF receptor 1. Expressed on some tumor cells but also on suppressive immune cells, including regulatory T cells and myeloid-derived suppressor cells [75].
*TPR*	Nucleoprotein TPR	Implicated in a variety of nuclear functions, including nuclear transport, chromatin organization, regulation of transcription, and mitosis. More recently, Tpr function has been linked to events including p53 signaling [76].

Note: Proteins filtered based on mascot score >10, ≥ unique peptides, high abundance or peak found in all biological and technical replicates, and high FDR protein confidence (99%).

**Table 7:** Details of proteins that have been reported in other cancers and that were exclusively found in the 10 K pellets of all five mesothelioma cell lines while being absent in the control cell line.

Gene	Protein Name	Description and Reports in Cancer
*AURKB*	Aurora kinase B	Dominant-negative effect on cytokinesis. Overexpression found in lung cancer, leukemia, and prostate cancer.
*EPHB2*	Ephrin type-B receptor 2	May be involved in disease pathogenesis. EPHB2 mutations have been found in a prostate cancer cell line derived from a brain metastasis. Also found in bleeding disorders, platelet-type, characterized by increased bleeding tendency due to platelet dysfunction.
*ERCC4*	DNA repair endonuclease XPF	Associated with hypersensitivity to DNA-damaging agents, chromosomal instability (increased chromosome breakage), and defective DNA repair.
*FAS*	Fas cell surface death receptor	A death receptor and a member of the tumor necrosis factor-receptor superfamily (CD antigen CD95). Fas ligand plays a key role in apoptotic signaling pathways. Mutations can prevent the immune system from attacking tumor cells.
*HRAS*	GTPase HRas	Involved in regulating cell division. Mutations in HRAS are implicated in a variety of human tumors such as thyroid cancer and bladder cancer.
*KCTD7*	Potassium channel tetramerization domain containing 7	Protein coding gene with features of a tumor suppressor. Found in brain cancer.
*MAD1L1*	Mitotic spindle assembly checkpoint protein MAD1	A checkpoint gene, where its dysfunction is associated with chromosomal instability. Mutations in MAD1L1 reported in colon and lung cancers.
*MSH2*	DNA mismatch repair protein Msh2	Tumor suppressing gene involved in DNA repair. Found in hereditary non-polyposis colorectal cancer 1, Muir-Torre syndrome, Endometrial cancer, Mismatch repair cancer syndrome 2, Colorectal cancer.
*MT-ATP8*	ATP synthase protein 8	Protein coding gene. Mutations found in breast, ovarian, cervical, and thyroid cancers.
*NDUFA13*	NADH:ubiquinone oxidoreductase subunit A13	Protein coding gene. Can function as a tumor suppressor. Found in hurthle cell thyroid carcinoma.
*PALLD*	Palladin (Sarcoma antigen NY-SAR-77)	A component of actin-containing microfilaments that control cell shape, adhesion, and contraction. Found in pancreatic cancer 1.
*PTK2*	Focal adhesion kinase 1 (FADK 1)	Aberrant PTK2/FAK1 expression may play a role in cancer cell proliferation, migration, and invasion, in tumor formation and metastasis. PTK2/FAK1 overexpression is seen in breast, ovarian, colorectal, and lung cancers.
*RHBDF2*	Inactive rhomboid protein 2	A protein coding gene. Found in esophageal cancer.
*RYR1*	Ryanodine receptor 1	A major gatekeeper of the calcium channel in skeletal muscle. Found in malignant hyperthermia 1.
*SRGAP1*	SLIT-ROBO Rho GTPase-activating protein 1	A protein coding gene. Found in thyroid cancer.
*TCF7L2*	Transcription factor 7-like 2	A transcription factor in the Wnt-signaling pathway. Found in colorectal cancer.
*WNT10A*	Protein Wnt-10a	Part of a large family of WNT genes, which are protein coding genes involved in Wnt signaling that regulate the interactions between cells during embryonic development. Found in colorectal cancer.

**Table 8:** Details of proteins that have been reported in other cancers and that were exclusively found in the 18 K pellets of all five mesothelioma cell lines while being absent in the control cell line.

*Gene*	Protein Name	Description and Reports in Cancer
*EHBP1*	EH domain-binding protein 1	A protein coding gene associated with endocytic trafficking. Found in prostate cancer.
*LIFR*	Leukemia inhibitory factor receptor	Mediates the action of the leukemia inhibitory factor that is involved in cellular differentiation, proliferation, and survival in the adult and the embryo. Found in epithelial tumors of the salivary gland.

**Table 9:** Details of proteins that have been reported in other cancers and that were exclusively found in the 100 K pellets of all five mesothelioma cell lines while being absent in the control cell line.

*Gene*	Protein Name	Description and Reports in Cancer
*DPOD1*	DNA polymerase delta catalytic subunit	Found in colorectal cancer.
*UHRF2*	E3 ubiquitin-protein ligase UHRF2	DNA copy number loss is found in multiple kinds of malignancies including brain, breast, stomach, kidney, hematopoietic tissue, and lung cancers.
*FANCJ*	Fanconi anemia group J protein	Associated with hypersensitivity to DNA-damaging agents, chromosomal instability (increased chromosome breakage), and defective DNA repair. Found in breast cancer.
*NSD1*	Nuclear receptor binding SET domain protein 1	The NSD1 enzyme controls the activity of genes involved in normal growth and development. Found in childhood acute myeloid leukemia, neuroblastoma, and glioma.
*NOTCH3*	Notch receptor 3	Plays a key role in the function and survival of vascular smooth muscle cells, and for the maintenance of blood vessels, including those that supply blood to the brain. Found to have oncogenic and tumor suppressive roles in various cancers, including breast, colorectal, lung, prostate, and ovarian cancers. Also found in myofibromatosis, infantile 2.
*STK10*	Serine/threonine-protein kinase 10	Functions as a tumor suppressor. Dysfunction of STK10 activity can promote anti-apoptotic effects, contributing to carcinogenesis. Found in peripheral T-cell lymphoma and testicular germ cell tumor.
*SAMD9*	Sterile alpha motif domain-containing protein 9	Play a role in regulating cell proliferation and apoptosis. Mutations can suppress cell cycle. Reported in tumoral calcinosis and acute myelogenous leukemia.
*JAK2*	Janus kinase 2	Plays a central role in cytokine and growth factor signaling. Growth factors like TGF-beta 1 induce phosphorylation and activation of this kinase to the nucleus, where they influence gene transcription. This gene is a downstream target of the pleiotropic cytokine IL6 that is produced by B cells, T cells, dendritic cells, and macrophages to produce an immune response or inflammation. Dysregulation of the IL6/JAK2/STAT3 signaling pathways produces increased cellular proliferation and neoplasms of hematopoietic stem cells. Reported in leukemia.

**Table 10:** Expression of biomarkers known to have biomarker potential in pleural mesothelioma.

Protein Name	Gene	H28			VMC23			H226			MM05			MSTO		
		10 K	18 K	100 K	10 K	18 K	100 K	10 K	18 K	100 K	10 K	18 K	100 K	10 K	18 K	100 K
Mesothelin *	*MSLN*							+	+	+	+	+	+	+	+	+
Programmed cell death 1 ligand 2 (PD-L2)	*PDCD1LG2*				+						+					
Bridging Integrator 1	*BIN1*		-			-	+		-			-			-	
Osteopontin	*SPP1*	+	+	+							+			+		+
Fibulin-3	*EFEMP1*	-	-	-	-	-										
High mobility group protein B1	*HMGB1*			-		-	-	-	-				-			
Calretinin *	*CALB2*							+	+	+						
Calreticulin *	*CALR*	•	•		•	•	•	•	•	•	•	•	•	•	•	•
Vascular endothelial growth factor A	*VEGFA*	+	+	+												+
Cyclin-dependent kinase inhibitor 2A	*CDKN2A*	-			-			-			-			-		
Lysyl oxidase homologs 1	*LOXL1*				+	+	+	+	+	+	+	+	+	+	+	+
Thrombospondin-2	*THBS2*	-	-				+			+			+			+
Cadherin-11	*CDH11*	+			+	+	+	+	+	+			+			
Desmin	*DESM*											+				
GLUT-1	*SLC2A1*			+				-				+	+		+	
Cellular tumor antigen p53	*TP53*	-			-			-			-			-		
E3 ubiquitin-protein ligase XIAP	*XIAP*															
Neural cell adhesion molecule 1	*NCAM*							-								
Tumor necrosis factor alpha-induced protein 2 *	*TNFAIP2*															+
Tumor necrosis factor receptor superfamily member 11B *	*TNFRSF11B*										+	+	+			
Tumor necrosis factor receptor superfamily member 6 *	*FAS*				+			+								
Vesicle transport protein GOT1B *	*GOLT1B*				+											
Galectin-1 *	*LGALS1*	•	•	•	•	•	•	•	•	•	•	•	•	•	•	•
Superoxide dismutase 1 *	*SOD1*	•		-	•	•	•	•	•	•	•	•	•	•	•	•
Superoxide dismutase 2 *	*SOD2*	•	-	-	•	•	•	•	•	•	•	•	•	•	•	
Signal transducer and activator of transcription 1-alpha/beta *	*STAT1*		+				+			+				-		
Signal transducer and activator of transcription *	*STAT3*				+	+	+	+	+	+						
Translationally controlled tumor protein *	*TPT1*	•			•	•	•	•	•	•	•	•	•	•	•	•
Vimentin *	*VIM*	•	•	•	•	•	•	•	•	•	•	•	•	•	•	•

* Mesothelioma-associated protein cargo also found in exosomes by Greening et al. 2016 [90]. Note: ‘+’ indicates overexpression of protein relative to the corresponding EV type of the control cell line, MeT-5A; ‘-‘ indicates downregulation of protein relative to the corresponding EV type of the control cell line, MeT-5A. Data are based on the log-fold change. • denotes high abundance found in samples without differential expression analysis.

## Data Availability

The data presented in this study are available on request from the corresponding author.

## Supplementary Materials

The following supporting information can be downloaded at: https://www.mdpi.com/article/10.3390/cancers15082364/s1. Figure S1: Western blot results for all markers in the large and small EV fractions across all cell lines used in the study. Table S1. Common proteins from this study and from mEXOS signature from Greening et al. (2016). File S1: Original blot.
