# Supplementary material for: Small and Large Extracellular Vesicles Derived from Pleural Mesothelioma Cell Lines Offer Biomarker Potential
Source: Cancers (Basel). 2023 Apr 18;15(8):2364. doi: 10.3390/cancers15082364 (PMC10136721; doi:10.3390/cancers15082364)
Supplement: Supplementary file 1 [file cancers-15-02364-s001.zip › cancers-2227141 supplementary table.pdf]

Supplementary Table

Table S1. Common proteins from this study and from mEXOS signature from Greening *et al.* (2016).

Proteins	Gene	10 K	18 K	100 K
Alpha-actinin-4 (F-actin cross-linking protein) (Non-muscle alpha-acti	ACTN4	5	5	5
Alpha-enolase (EC 4.2.1.11) (2-phospho-D- glycerate hydro-lyase) (C-myc	ENO1	5	5	5
Fibronectin	FN1	5	5	5
Glyceraldehyde-3-phosphate dehydrogenase (GAPDH) (EC 1.2.1.12) (Peptid	GAPDH	5	5	5
Heat shock 70 kDa protein 1A/1B (Heat shock 70 kDa protein 1/2) (HSP70	HSPA1A	5	5	5
Heat shock cognate 71 kDa protein (Heat shock 70 kDa protein 8)	HSPA8	5	5	5
Heat shock protein HSP 90-alpha (Heat shock 86 kDa) (HSP 86) (HSP86)	HSP90AA1	5	5	5
Heat shock protein HSP 90-beta (HSP 90) (Heat shock 84 kDa) (HSP 84)	HSP90AB1	5	5	5
Myosin-9 (Cellular myosin heavy chain, type A) (Myosin heavy chain 9)	MYH9	5	5	5
Pyruvate kinase PKM (EC 2.7.1.40) (Cytosolic thyroid hormone-binding p	PKM	5	5	5
Annexin A1 (Annexin I) (Annexin-1) (Calpactin II) (Calpactin-2) (Chrom	ANXA1	5	5	4
Annexin A5 (Anchorin CII) (Annexin V) (Annexin-5) (Calphobindin I) (CB	ANXA5	5	5	4
Integrin alpha-3 (CD49 antigen-like family member C) (FRP-2) (Galactop	ITGA3	5	5	4
Moesin (Membrane-organizing extension spike protein)	MSN	5	5	4
Annexin A2 (Annexin II) (Annexin-2) (Calpactin I heavy chain) (Calpact	ANXA2	5	4	4
Laminin subunit alpha-1 (Laminin A chain) (Laminin-1 subunit alpha) (L	LAMA1	4	3	4
EGF-like repeat and discoidin I-like domain-containing protein 3 (Deve	EDIL3	4	2	4
Annexin A6	ANXA6	4	1	0
Nidogen-1 (NID-1) (Entactin)	NID1	3	4	4
Complement factor B (Uncharacterized protein) (cDNA FLJ55673, highly s	CFB	3	3	2
Fibrillin-2	FBN2	3	3	2
Branched-chain-amino-acid aminotransferase, cytosolic (BCAT(c)) (EC 2.	BCAT1	3	2	3
tRNA-splicing ligase RtcB homolog (EC 6.5.1.3)	RTCB	3	2	3
Vitamin K-dependent protein S	PROS1	3	1	2
Procollagen galactosyltransferase 1 (EC 2.4.1.50) (Collagen beta(1-O)g	COLGALT1	3	0	1
CD63 antigen	CD63	2	3	4
Pregnancy-specific beta-1-glycoprotein 4 (PS-beta-G-4) (PSBG-4) (Pregn	PSG4	2	2	2
Pappalysin-1 (EC 3.4.24.79) (Insulin-like growth factor-dependent IGF-	PAPPA	2	1	3
2',3'-cyclic-nucleotide 3'-phosphodiesterase (CNP) (CNPase) (EC 3.1.4.	CNP	2	1	1
Clustered mitochondria protein homolog	CLUH	1	1	2
Intelectin-1 (ITLN-1) (Endothelial lectin HL-1) (Galactofuranose-bindi	ITLN1	1	1	2
Calretinin (CR) (29 kDa calbindin)	CALB2	1	1	1
Galactosylceramide sulfotransferase (Fragment)	GAL3ST1	1	1	1
Integrin alpha-4 (CD49 antigen-like family member D) (Integrin alpha-I	ITGA4	1	1	1
Interferon-induced protein with tetratricopeptide repeats 1 (IFIT-1) (	IFIT1	1	1	1
Interferon-induced protein with tetratricopeptide repeats 2 (IFIT-2) (	IFIT2	1	1	1
Oncostatin-M-specific receptor subunit beta (Interleukin-31 receptor s	OSMR	1	1	1
Platelet-derived growth factor D (PDGF-D) (Iris-expressed growth facto	PDGFD	1	1	1
Teneurin-2	TENM2	1	1	1
Carboxypeptidase E (CPE) (EC 3.4.17.10) (Carboxypeptidase H) (CPH) (En	CPE	1	1	0
Neuropilin 2	NRP2	1	0	3
A disintegrin and metalloproteinase with thrombospondin motifs 12 (ADA	ADAMTS12	1	0	2
Interleukin-7 receptor subunit alpha	IL7R	1	0	2
2'-5'-oligoadenylate synthase-like protein (2'-5'-OAS-related protein)	OASL	1	0	0
Adipocyte plasma membrane-associated protein (Protein BSCv)	APMAP	1	0	0
ADP-ribosyl cyclase 2	BST1	0	1	1
Desmoplakin (DP) (250/210 kDa paraneoplastic pemphigus antigen)	DSP	0	1	1
Heterogeneous nuclear ribonucleoprotein D-like (hnRNP D-like) (hnRNP D)	HNRNPDL	0	0	1
