# Supplementary material for: Small and Large Extracellular Vesicles Derived from Pleural Mesothelioma Cell Lines Offer Biomarker Potential
Source: Cancers (Basel). 2023 Apr 18;15(8):2364. doi: 10.3390/cancers15082364 (PMC10136721; doi:10.3390/cancers15082364)
Supplement: Supplementary file 1 [file cancers-15-02364-s001.zip › cancers-2227141-supplementary figure.pdf]

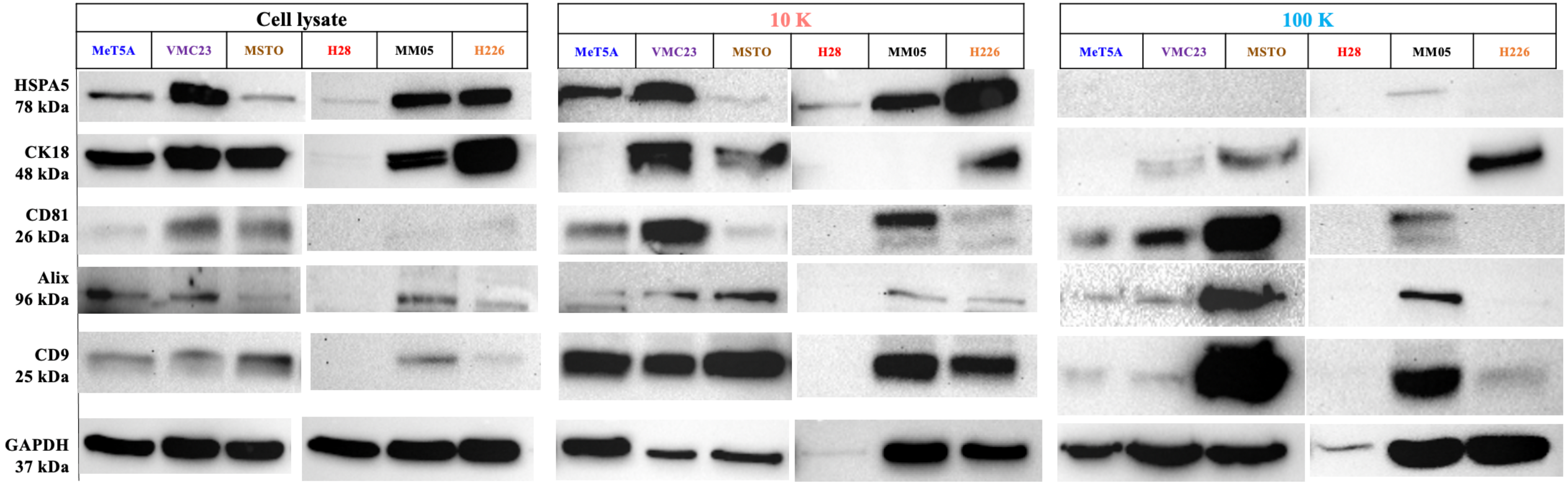

Figure S1: Western blot results for all markers in the large and small EV fractions across all cell lines used in the study.
