# Supplementary material for: Small and Large Extracellular Vesicles Derived from Pleural Mesothelioma Cell Lines Offer Biomarker Potential
Source: Cancers (Basel). 2023 Apr 18;15(8):2364. doi: 10.3390/cancers15082364 (PMC10136721; doi:10.3390/cancers15082364)
Supplement: Supplementary file 1 [file cancers-15-02364-s001.zip › Supplementary file S1_WB uncropped images_v2.pdf]

Supplementary Data

- Data shown are uncropped western blot images.
- Please note that with antibodies which had a large difference in their molecular weights, the membranes were imaged with two antibodies as follows:

1. Membrane was incubated with the first antibody.
2. Membrane was washed, stained and imaged, as described in the Methods section, to obtain the images for the first antibody.
3. Membrane was then washed again three times and incubated with the second antibody.
4. Membrane was washed three times, stained, and imaged again to obtain images for the second antibody.

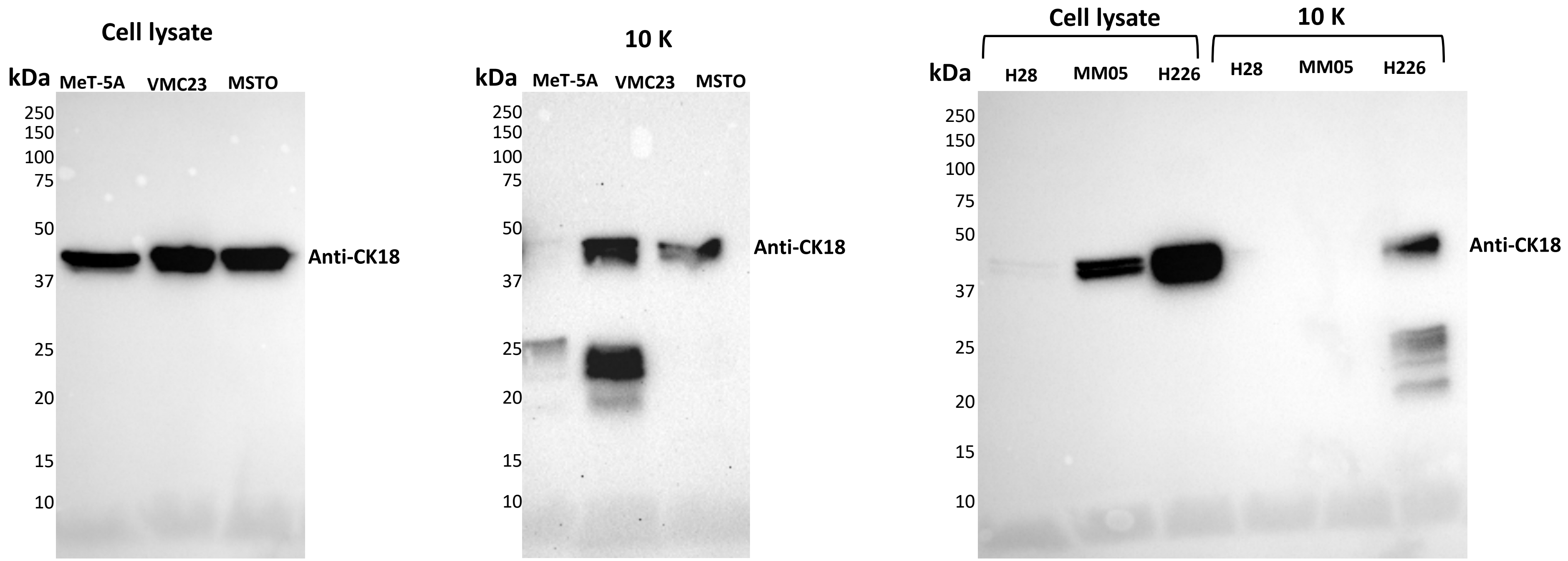

Membranes above were washed, incubated with anti-BiP, and then imaged again as shown below.

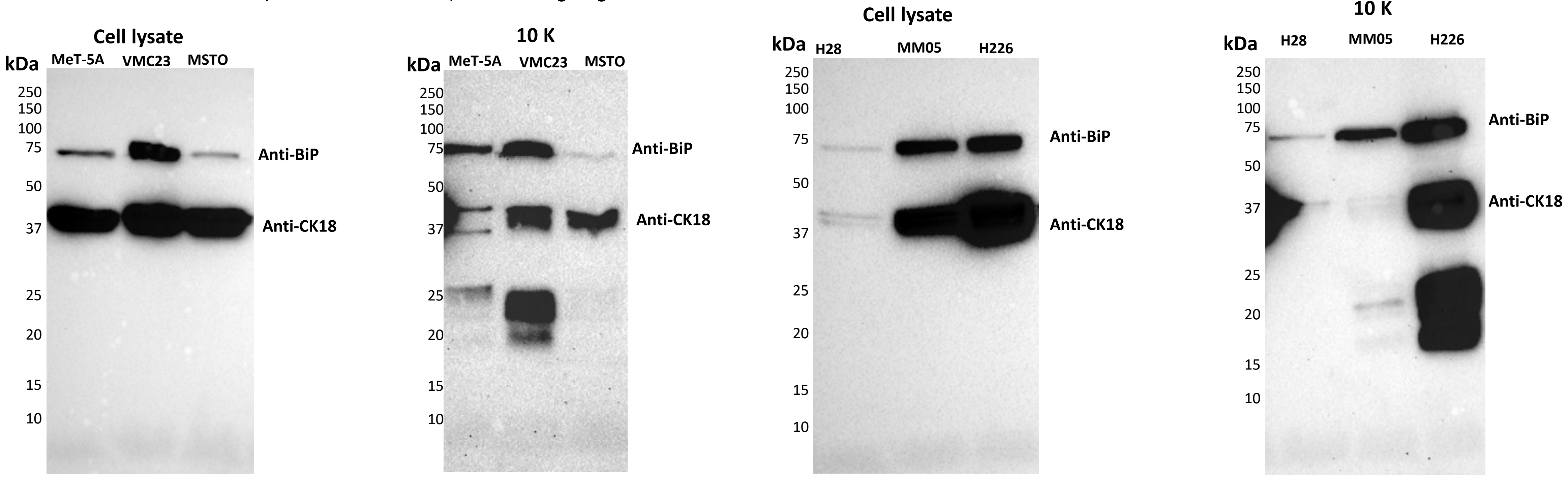

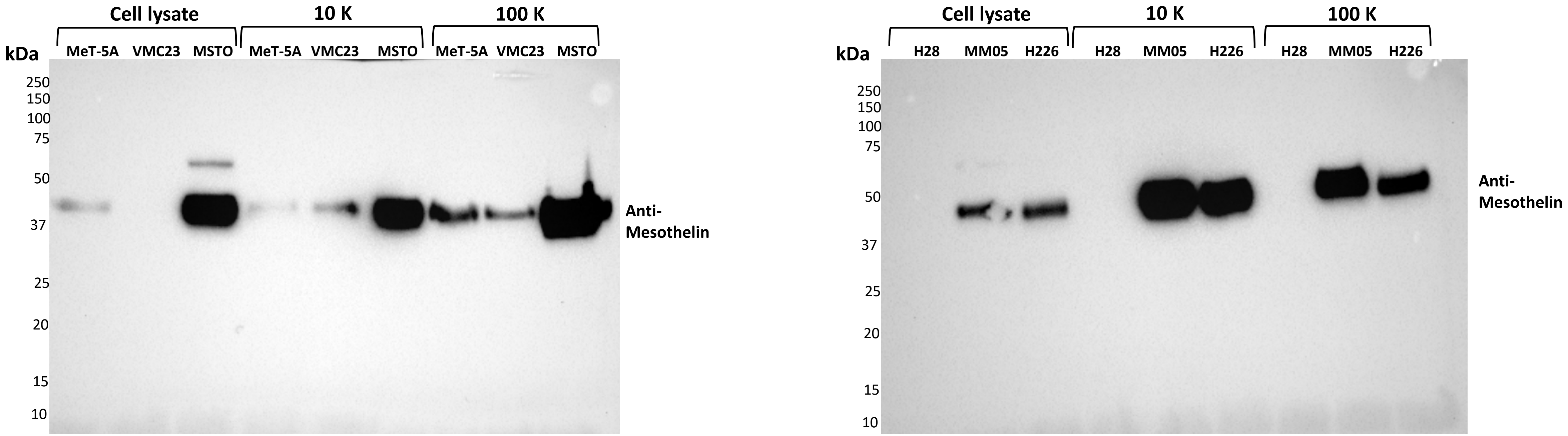

Membranes above were washed, incubated with anti-CD81, and then imaged again as shown below.

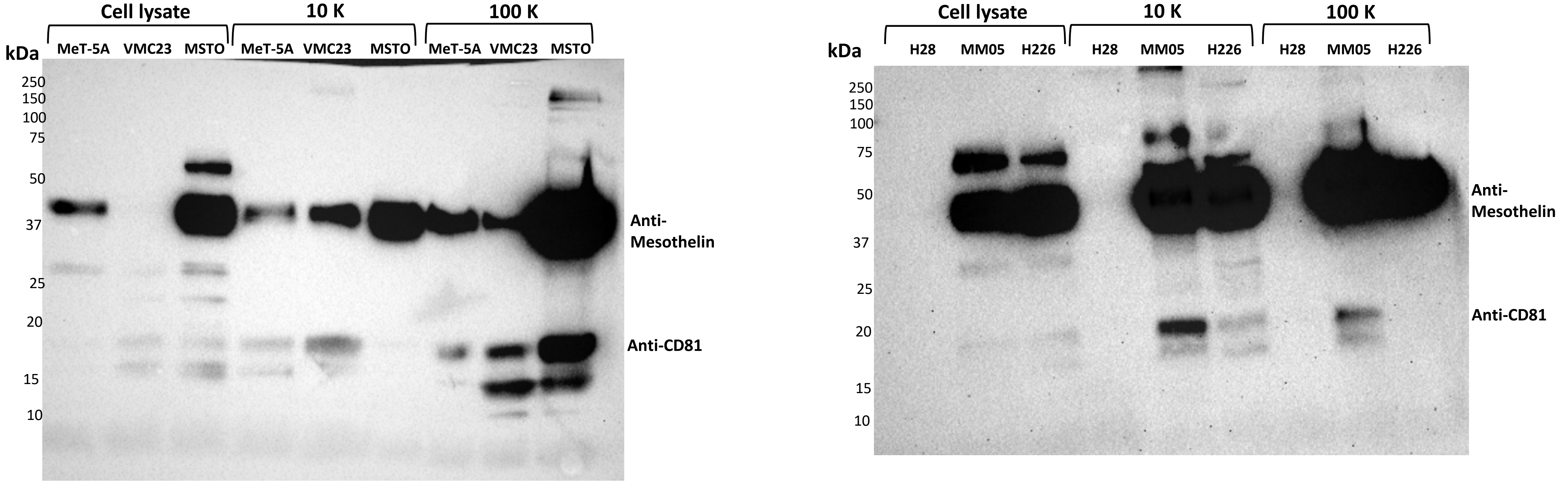

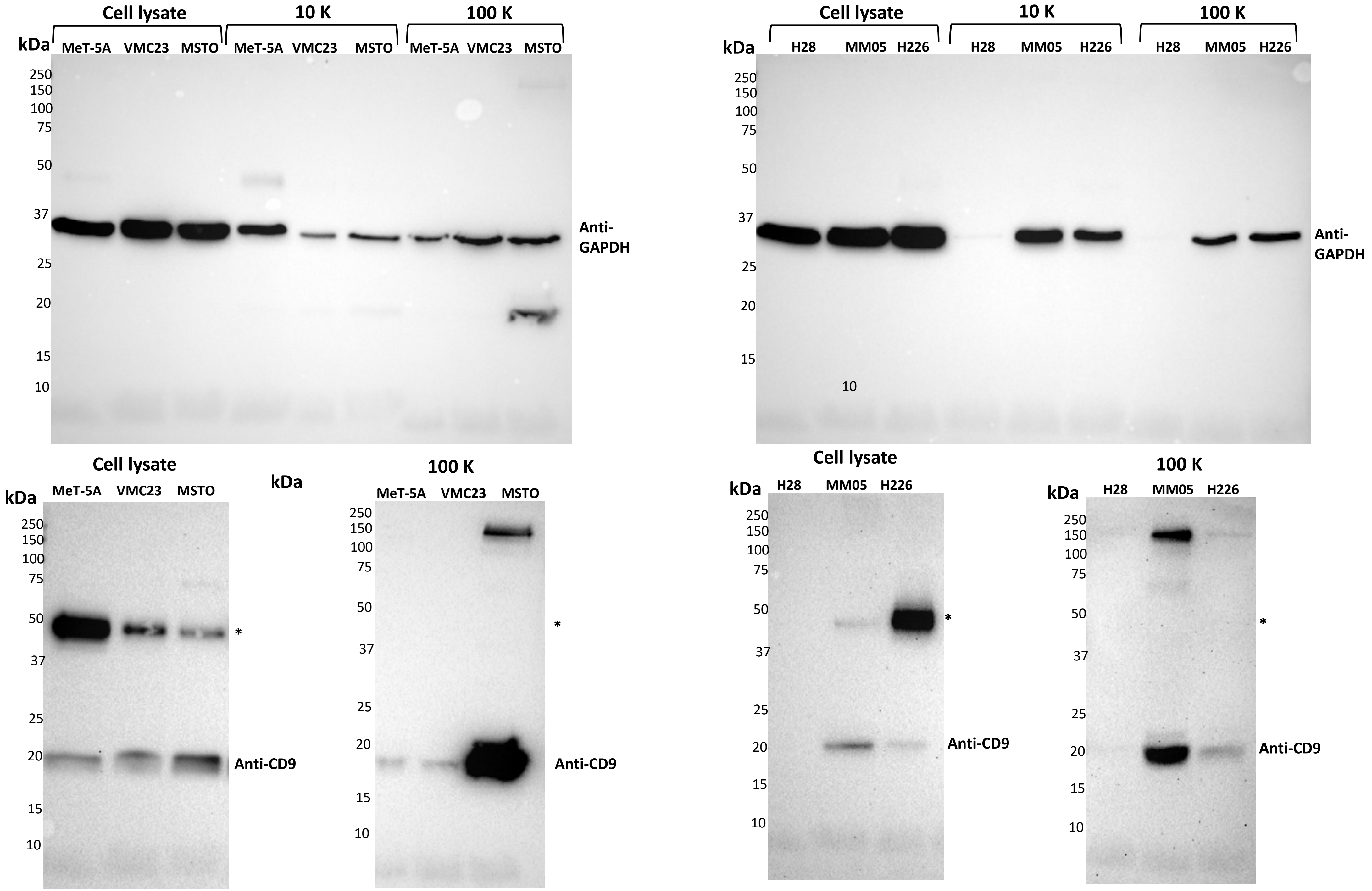
Cell lysate
10 K
100 K
kDa
MeT-5A
VMC23
MSTO
MeT-5A
VMC23
MSTO
MeT-5A
VMC23
MSTO
250
150
100
75
50
37
25
20
15
10
Anti-GAPDH
Cell lysate
10 K
100 K
kDa
H28
MM05
H226
H28
MM05
H226
H28
MM05
H226
250
150
100
75
50
37
25
20
15
10
Anti-GAPDH
Cell lysate
kDa
MeT-5A
VMC23
MSTO
250
150
100
75
50
37
25
20
15
10
*
Anti-CD9
100 K
kDa
MeT-5A
VMC23
MSTO
250
150
100
75
50
37
25
20
15
10
*
Anti-CD9
Cell lysate
kDa
H28
MM05
H226
250
150
100
75
50
37
25
20
15
10
*
Anti-CD9
100 K
kDa
H28
MM05
H226
250
150
100
75
50
37
25
20
15
10
*
Anti-CD9

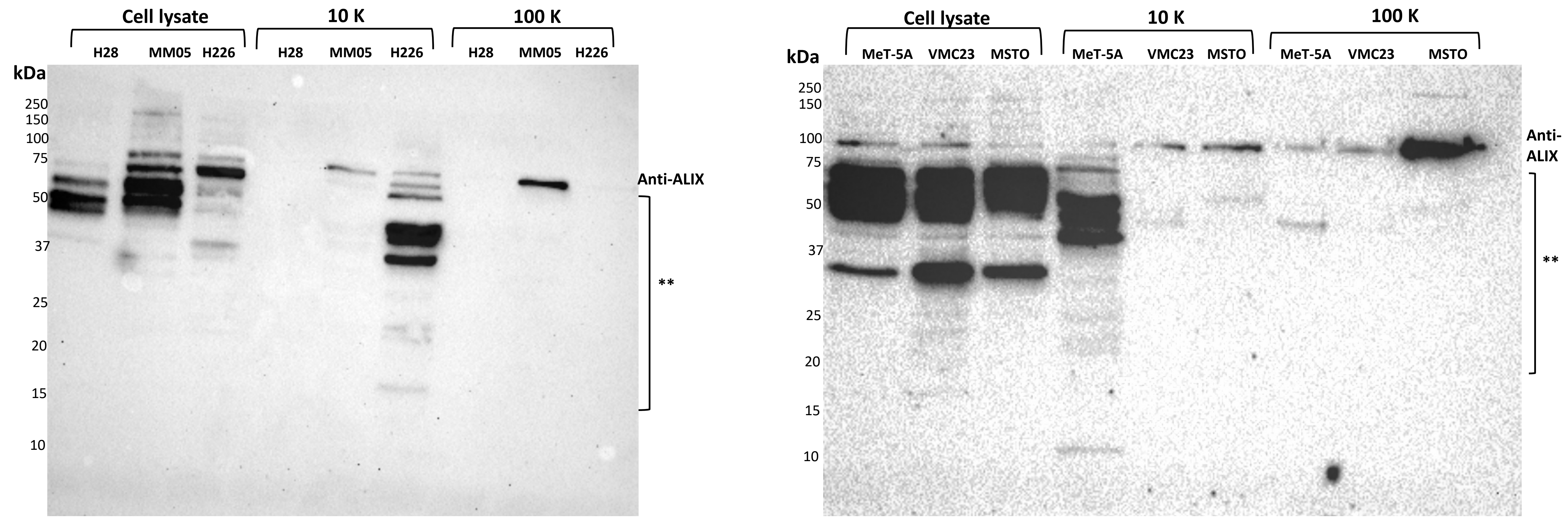

***Membrane was first incubated and imaged with a monoclonal antibody that will not be identified as it is outside the scope of this manuscript.**

**** Membrane was first incubated and imaged with a polyclonal antibody that will not be identified as it is outside the scope of this manuscript.**

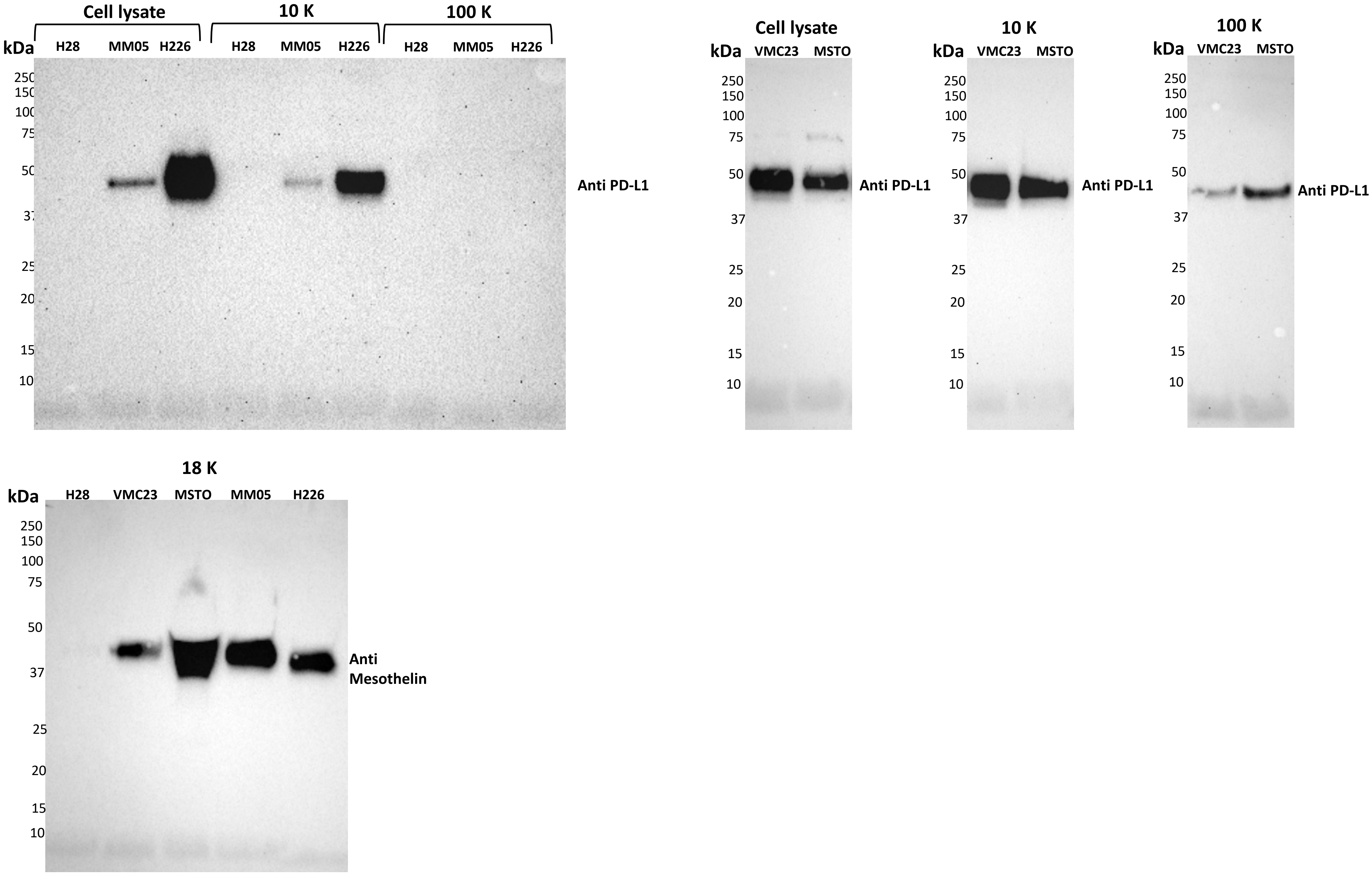
Cell lysate
10 K
100 K
kDa
H28
MM05
H226
H28
MM05
H226
H28
MM05
H226
250
150
100
75
50
37
25
20
15
10
Anti PD-L1
Cell lysate
kDa
VMC23
MSTO
250
150
100
75
50
37
25
20
15
10
Anti PD-L1
10 K
kDa
VMC23
MSTO
250
150
100
75
50
37
25
20
15
10
Anti PD-L1
100 K
kDa
VMC23
MSTO
250
150
100
75
50
37
25
20
15
10
Anti PD-L1
18 K
kDa
H28
VMC23
MSTO
MM05
H226
250
150
100
75
50
37
25
20
15
10
Anti
Mesothelin
